# Waves of attention: patterns and themes of international antimicrobial resistance reports, 1945–2020

**DOI:** 10.1136/bmjgh-2021-006909

**Published:** 2021-11-05

**Authors:** Kristen Overton, Nicolas Fortané, Alex Broom, Stephanie Raymond, Christoph Gradmann, Ebiowei Samuel F Orubu, Scott H Podolsky, Susan Rogers Van Katwyk, Muhammad H Zaman, Claas Kirchhelle

**Affiliations:** 1 Infectious Diseases Department, Prince of Wales Hospital Randwick, Sydney, New South Wales, Australia; 2 Centre for Social Research in Health, University of New South Wales, Sydney, New South Wales, Australia; 3 IRISSO, CNRS, INRAE, Paris-Dauphine University, PSL, Paris, France; 4 School of Social and Political Sciences, Sydney Centre for Healthy Societies, The University of Sydney, Sydney, New South Wales, Australia; 5 Institute of Health and Society, Department of Community Medicine and Global Health, University of Oslo, Oslo, Norway; 6 Institute for Health System Innovation and Policy, Boston University, Boston, Massachusetts, USA; 7 Department of Biomedical Engineering, Boston University, Boston, Massachusetts, USA; 8 Department of Global Health and Social Medicine, Harvard Medical School, Boston, Massachusetts, USA; 9 Global Strategy Lab, York University, Toronto, Ontario, Canada; 10 Department of International Health, Boston University, Boston, Massachusetts, USA; 11 School of History, University College Dublin, Dublin, Ireland

**Keywords:** infections, diseases, disorders, injuries, public Health, health policies and all other topics

## Abstract

This article uses quantitative and qualitative approaches to review 75 years of international policy reports on antimicrobial resistance (AMR). Our review of 248 policy reports and expert consultation revealed waves of political attention and repeated reframings of AMR as a policy object. AMR emerged as an object of international policy-making during the 1990s. Until then, AMR was primarily defined as a challenge of human and agricultural domains within the Global North that could be overcome via ‘rational’ drug use and selective restrictions. While a growing number of reports jointly addressed human and agricultural AMR selection, international organisations (IOs) initially focused on whistleblowing and reviewing data. Since 2000, there has been a marked shift in the ecological and geographic focus of AMR risk scenarios. The Global South and One Health (OH) emerged as foci of AMR reports. Using the deterritorialised language of OH to frame AMR as a Southern risk made global stewardship meaningful to donors and legitimised pressure on low-income and middle-income countries to adopt Northern stewardship and surveillance frameworks. It also enabled IOs to move from whistleblowing to managing governance frameworks for antibiotic stewardship. Although the environmental OH domain remains neglected, realisation of the complexity of necessary interventions has increased the range of topics targeted by international action plans. Investment nonetheless continues to focus on biomedical innovation and tends to leave aside broader socioeconomic issues. Better knowledge of how AMR framings have evolved is key to broadening participation in international stewardship going forward.

**Key questions**

**What is already known?**

- The last two decades have seen a marked rise of attention to antimicrobial resistance (AMR) at the international political level.
- There have been multiple framings of AMR as a target for political intervention since the 1950s.

**What are the new findings?**

- Our analysis of 248 historical international and regional AMR policy documents and expert consultation revealed waves of political attention and the emergence of AMR as a sustained target for intervention at the international level during the 1990s.
- Since around 2000, the rise of new concepts like stewardship, the ‘One Health, One World’ paradigm and the ‘Global South’ has been accompanied by a shift of international policy-making from whistleblowing to governance.
- Our analysis highlights persistent biases such as a Northern dominance of report authorship, a deterritorialisation of stewardship policies regarding the Global South, and a neglect of the environmental One Health domain.

**What do the new findings imply?**

- We argue that critical engagement with how AMR is framed is crucial to developing more effective international engagement and maintaining political momentum for reform amidst declining attention.
- Defining AMR as a threat to Northern biosecurity risks ignoring important Southern perspectives and prioritising regional over planetary solutions.

## Introduction

The last decade has seen unprecedented international efforts to address the global threat of antimicrobial resistance (AMR). A highpoint of attention was reached with the announcement of the WHO’s 2015 Global Action Plan.^1^ However, even before the SARS-CoV-2 pandemic, international attention to AMR had begun to wane. Understanding the reasons for this decline, and the way the AMR problem has been framed by reports and decision-makers at the international level, is important for the long-term success of initiatives to preserve antibiotic efficacy and for maintaining pan-national investment in AMR solutions. In this paper, we combine quantitative and qualitative approaches from history and the social sciences to analyse the patterns and content of international AMR reporting. Our analysis of antibiotic politics at the international level both goes beyond existing historical studies’ focus on the Global North^2–4^ and also highlights the importance of policy documents in reflecting and shaping problem framings, political discourse and regulatory action.

We show that understandings of AMR, and which areas were included in policy-making, have evolved substantially. Between the 1950s and 1980s, AMR was primarily defined as a Northern challenge that could be overcome via ‘rational drug’ use or selective drug restrictions. It was only during the 1990s that AMR emerged as a consistent focus of international reporting and that a growing number of reports jointly addressed human and agricultural AMR selection. Starting in the new millennium and accelerating after 2010, the Global South and One Health (OH) emerged as key foci in international reporting. Framing of AMR as a Southern risk has led to a deterritorialisation of international policy discourse and growing pressure on low-income and middle-income countries (LMICs) to implement AMR-focused reforms. It has also boosted the status of international organisations (IOs) like the WHO, who have no longer limited themselves to whistleblowing but assumed active governance roles within global stewardship and surveillance frameworks. Calls for interventions are now couched in the language of ‘One World, One Health’—although the environmental domain remains neglected. Realisation of the complexity of necessary interventions has also led to an increase in the range of topics being targeted by international action plans. Despite acknowledging the structural complexities of AMR, the international community has focused investment on biomedical innovation (i.e., the therapeutics, diagnostics and stewardship trifecta) and tends to leave aside broader socioeconomic issues related to food and health systems and pharmaceutical markets. Finally, our analysis sheds light on international governance as such, in particular how new actors and power dynamics emerged over time.

Box 1 **Summary of key terms**

**Global North/South**

Global North primarily refers to regions and countries within Europe, North America and parts of Asia, Latin America and Oceania that have high levels of wealth, living standards, technology and international influence. By contrast Global South refers to primarily low-income and middle-income and politically marginalised regions in Latin America, Asia, Africa and Oceania. Originating in work by Antonio Gramsci on regional relations within Italy, the terms North and South emphasise geopolitical power relations rather than the historically dominant focus on development and cultural difference. Similar to the core-periphery terminology, the terms also reference histories of colonialism, extraction and ongoing socioeconomic inequalities.^89^

**Governance**

Refers to the exercise of power in the management of economic, political and social resources through all processes of interaction including laws, norms, economic relations, language, etc. Governance can be exercised both by governmental and non-governmental organisations as well as through markets or networks.^90^

**High-income, middle-income and low-income countries**

Based on the World Bank’s ATLAS methods, high-income countries have a gross national income per capita of US $12 696 or more (2020), middle-income countries have income levels ranging from $1046 to $12 695, low-income countries have a gross national income per capita of US$1045 or less.^91^

**International organisations (IOs)**

The category of IOs refers both to organisations that were established by an instrument of international law and involve at least three members as well as to non-treaty-based state coalitions like the G7.^7^

**One Health**

One Health refers to collaborative efforts of multiple disciplines at different local, national and global levels to attain optimal health for humans, animals and the environment. The term rose to prominence in international discourse around 2000 but is rooted in older traditions of one medicine and tropical medicine.^25 39^

## Methods

Our analysis was based on two complementary but distinct approaches to identifying patterns of attention and discovering how AMR was framed over time; (a) *content analysis* of international and regional AMR policy documents published between 1945 and 2020; (b) a *consultative process* involving experts in the field of AMR, to identify policy documents perceived to have the greatest impact. We did not involve patients or members of the public in the design of the study.

### A) Survey of 75 years of AMR reporting

Global health politics are shaped by a multitude of governmental and non-governmental actors. Since the origins of the international health system around 1900, non-governmental organisations like the Rockefeller Foundation, the Wellcome Trust or the Bill and Melinda Gates Foundation have influenced health policies at the national and international levels.^5 6^ This is also true for antibiotic policy where NGOs have played an increasingly important role since the 1980s.^3 4^ Unfortunately, it is nearly impossible to capture resulting reports and policy documents in a systematic manner. Many organisations only engaged with AMR on an *ad hoc*, national or regional basis while others were only active for a brief time. Smaller NGOs—but also some of the largest international donors—do not have accessible archives, published reports are not systematically captured by online databases, and distinctions between publication formats (report, press briefing, etc.) are not always clear.

To maintain analytical cohesion and avoid biasing results towards a select group of NGOs whose archives are available online, we have focused on reports by IOs and transnational organisations like the European Union (EU) whose archives are fully accessible and span multiple decades. For the purposes of this paper, the category of IOs refers both to organisations that were established by an instrument of international law and involve at least three members as well as to non-treaty-based state coalitions like the G7.^7^ While we fully acknowledge the role of NGOs and other actors like individual governments in shaping international antibiotic policy, we only include reports by NGOs and individual governments that are identified as highly significant in the historical literature and by our experts (see part B).

The organisations whose reports have been included in our analysis are: Asia-Pacific Economic Cooperation (APEC), European Centre for Disease Control (ECDC), European Commission (EC), European Food Safety Authority (EFSA), European Medicines Agency (EMA), European Parliament (EP), EU, The Food and Agriculture Organisation (FAO), G7, G20, Organisation for Economic Co-operation and Development (OECD), Transatlantic Taskforce on Antimicrobial Resistance (TATFAR), United Nations (UN), UN International Children’s Emergency Fund (UNICEF), UN Interagency Coordination Group (IACG) on AMR, The World Bank, WHO and World Organisation for Animal Health (OIE).

Building on an initial overview presented by author CK at a multidisciplinary AMR workshop in 2019,^8^ our review identified policy documents that target AMR at the international or regional level and were written between 1945 and the end of 2020. Policy documents were identified by searching a variety of online databases. To account for changing terminology over time, we used the broad keywords ‘AMR’, ‘antimicrobial’ and ‘antibiotic’ and limited (by hand) to international and regional reports. Potential biases involved in using English search terms were limited as a result of IOs’ multilingual publication polices.

- First, databases of key organisations dealing with AMR were systematically searched, including WHO, FAO, OIE, IACG, CODEX, UN, OECD, World Bank, G7, G20, APEC, EU organisations (see above) and TATFAR websites.
- Second, to account for NGOs’ and individual government reports’ influence on international policy, further documents were identified by scanning the reference lists of the identified documents and adding reports by NGOs that were highlighted as influential by our expert consultative process (see part B).
- Third, to compensate for the temporal bias of digital repositories, we identified older non-digitised policy documents through the literature on the history of AMR^2–4 9–13^ and our expert group (see part B).
- Fourth, we applied inclusion/exclusion criteria to this data set: our policy documents include policy action plans and guidelines, meeting reports, legislative frameworks, surveillance reports and substantive stakeholder engagement (eg, stewardship guidance for farmers); they exclude original research (eg, experimental studies, etc.), simple communication materials (eg, popular AMR summaries), national legislation and intermediary policy documents such as meeting minutes or consultation summaries (for coding categories, see online supplemental one). The result is a data set of 248 AMR policy documents (online supplemental table 2) published between 1945 and 2020.
- Fifth, we cross-checked our data set with PubMed using the same search terms and limiting to government publications and technical reports (n=36). No additional reports were identified that fit our selection criteria and we noted that the vast majority of our identified reports were not listed in academic databases, which have informed previous research in this area.^14–16^

10.1136/bmjgh-2021-006909.supp1 Supplementary data

10.1136/bmjgh-2021-006909.supp2 Supplementary data

### Data processing

- Our coding evaluation framework was informed by methodologies adopted by Wernli *et al* and Ogyu *et al*^14 15^ and guided by research themes identified by Tompson and Chandler’s survey of social sciences approaches to AMR.^17^ Three questions guided our coding: (A) How has attention to AMR evolved over time? (B) Have the areas this attention focuses on changed? and (C) Have the geographies of AMR attention shifted?
- Resulting analytical categories included year, author, document type, target audience, orientation, geography, aim of policy and intervention called for (online supplemental table 1). Each document was coded manually according to these categories, which allowed basic statistical analysis on how our data set is distributed.
- The framework analysis of all included documents was undertaken by one author (KO) for consistency. The full list of variables and associated values can be found in online supplemental table 1.

### B) Consultative process: identifying additional policy documents and ranking reports

To identify additional key documents published by organisations that do not fall into the above categories (part A), we conducted a consultative process with relevant experts. Twenty-five experts were identified and contacted due to their work in critical areas: history of drug regulation and AMR; social sciences of AMR, antibiotic markets and legal frameworks; involvement in antibiotic-related policy-making at the national and international levels. Ten (40%) of the contacted experts participated in the subsequent three rounds of consultation.

Round One: experts were asked to identify the international reports they felt have had the greatest input on AMR/stewardship since 1945 (for consensus list, see online supplemental table 3). Results informed step 2 and 3 of part A (see above) and the creation of our data set of 248 reports (online supplemental table 2).

10.1136/bmjgh-2021-006909.supp3 Supplementary data

Round Two: a compilation of expert responses (online supplemental table 3) was circulated to the entire group, and via an online survey, we asked experts to rank the most influential top 10 of identified reports (figure 9). The survey provided an opportunity to comment on their ranking. Eight survey responses were received.

Round Three: we circulated the complete paper including the top 10 list and full policy reports and asked for comments on our findings. Respondents were invited to become coauthors on the final paper.

## Results

Our analysis revealed waves of international attention to AMR with new framings of AMR emerging over time. The decade after 2010 saw a peak of international activity and the publication of many of the most influential reports identified by our expert consultation. Our findings complement qualitative and quantitative evaluations of scientific reporting and national policy-making on AMR by other researchers^3 4 11–16^ but go beyond them in identifying: the origin of AMR as a focus of international policy-making; the significance of the simultaneous rise of themes like OH and the Global South and the accompanying shift of IOs’ role from whistleblowing to governance; and a decline of international attention prior to COVID-19.

### Identified AMR policy documents

**Figure 1:**
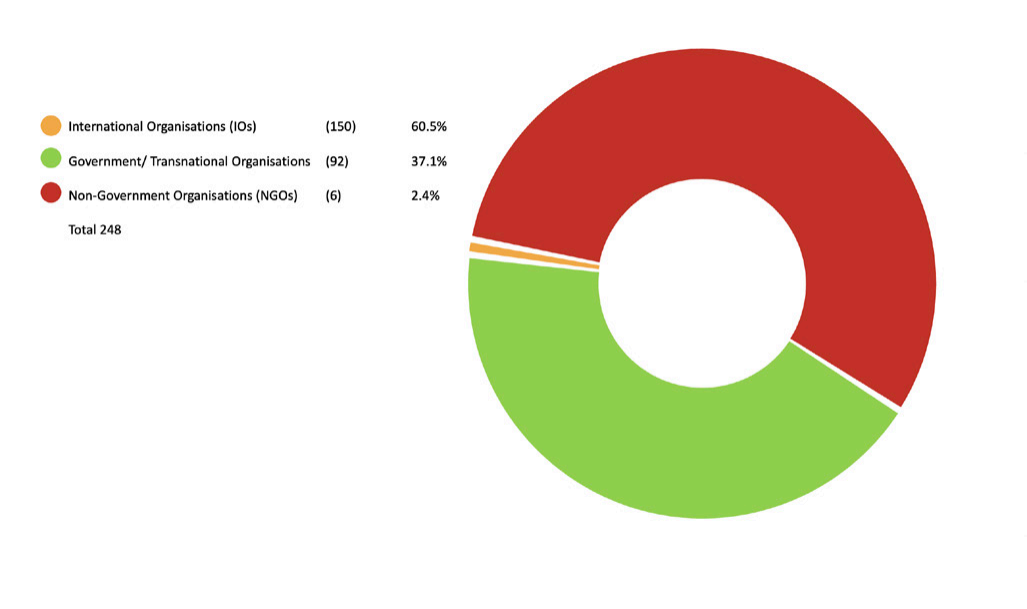
Publisher of report.

**Figure 2:**
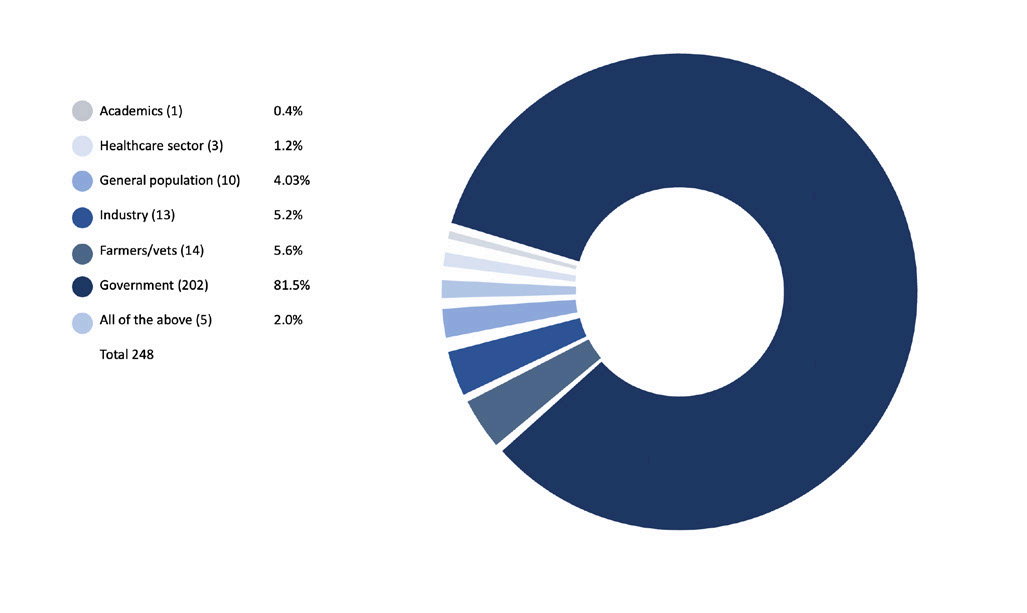
Target audiences for AMR reports.

**Figure 3:**
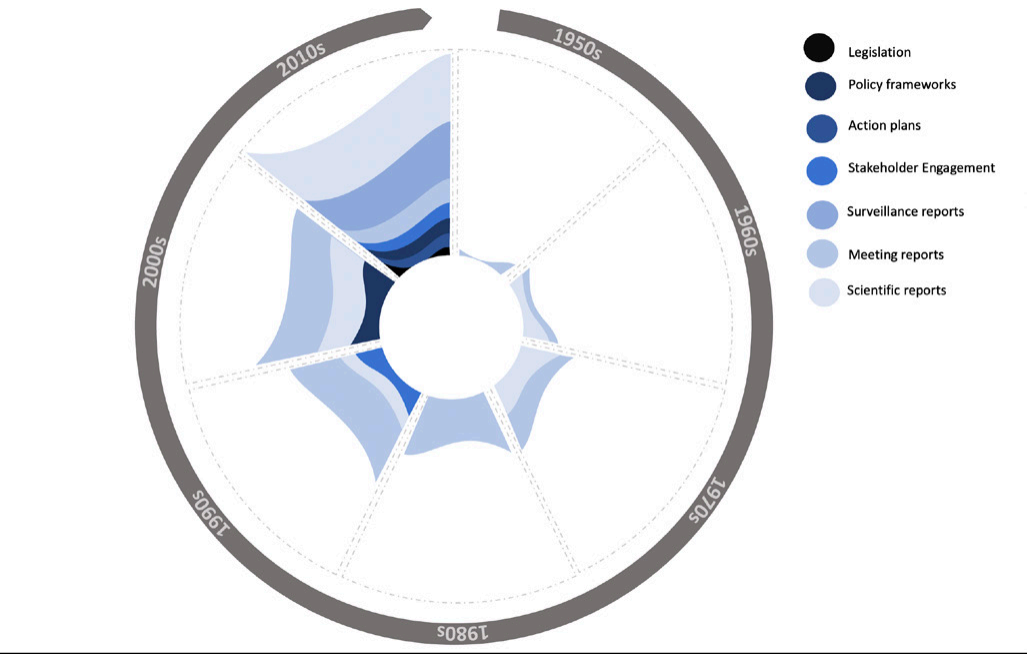
Types of AMR reports by decade (1950s–2010s).

Our survey of 75 years of reporting (part A) and additional expert consultation (part B) identified 248 international and regional AMR policy documents. The majority of policy documents were authored by IOs (n=150, 60.5%); followed by governments/transnational organisations (n=92, 37.1%); we included nine additional reports from NGOs (n=6, 2.4%) (see figure 1). Most policy documents targeted governments as the main audience (n=202, 81.5%) (see figure 2). In the IO category, the WHO was the largest producer of documents with 60 solo-authored and a further 21 coauthored reports, followed by FAO with 29 solo-authored and 20 coauthored documents and OIE with 11 solo-authored and 16 coauthored documents. Of the 248 documents, we coded 87 as scientific or technical reports, 60 as meeting reports, 47 as surveillance reports, 18 as stakeholder engagement (targeting public education), 16 as action plans, 13 as policy frameworks and 7 as legislation (See figure 3).

### Waves of attention

**Figure 4:**
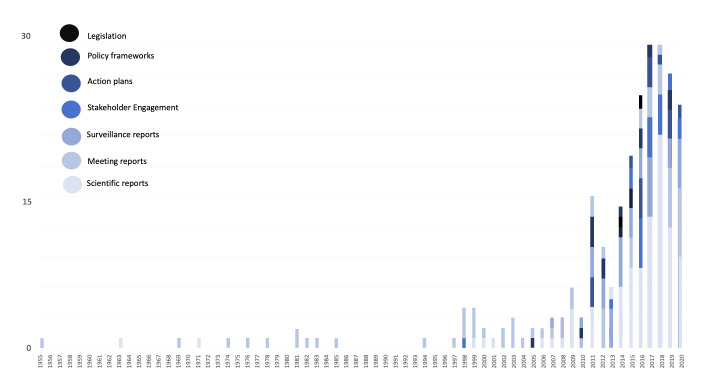
Types of AMR reports by year (1955–2020).

The first international report on AMR was published in 1955.^18^ Subsequent attention was inconsistent, with international reports reviewing AMR threats of individual—mostly agricultural—practices from the mid-1960s onwards. It was not until the early to mid-1990s when acknowledgement of AMR as an escalating (human) health concern triggered more consistent action. Attention regarding AMR at the international policy level has increased consistently since the late 1990s (see figure 4). This increase in policy push coincides with a planetary framing of AMR threats that have to be dealt with at the international level. Despite the ongoing escalation of the AMR problem,^19^ the number of international reports began to decrease after 2017/2018 (see figure 4). Alongside recent drops of international funding and support by important national donors,^20 21^ this may indicate that attention for AMR at the international level could have reached a turning point even before the COVID-19 pandemic.

### From whistleblowing to governance

Despite surges in AMR data from the early 1970s and mid-1990s onwards as well as individual 1990s national level action plans and an abortive WHO call to action in 2001,^9 11 12 22 23^ sustained and formalised international action only emerged in the 2010s in the form of iterative international and regional AMR policy documents (see figure 4). This observation correlates with changes in the structure of international governance of AMR. In the 1990s and 2000s, UN-level IOs like the WHO were mostly behaving as ‘whistle-blowers’ whose objective was to raise awareness and put AMR on the political agenda, while from the 2010s onwards, they have increasingly behaved like public authorities (in particular towards Southern states) in such a way that the documents they now publish produce ‘real’ action plans, guidelines, and surveillance infrastructures.

### Emergence of Global South out of Northern discussion

**Figure 5:**
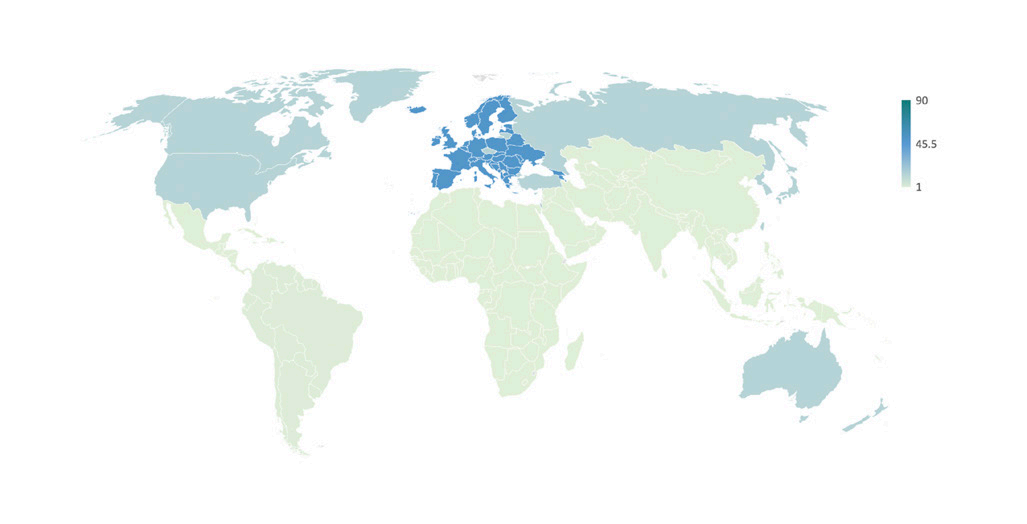
Total AMR reports by target locale* (1955–2000s). *Excluding 112 worldwide reports.

**Figure 6:**
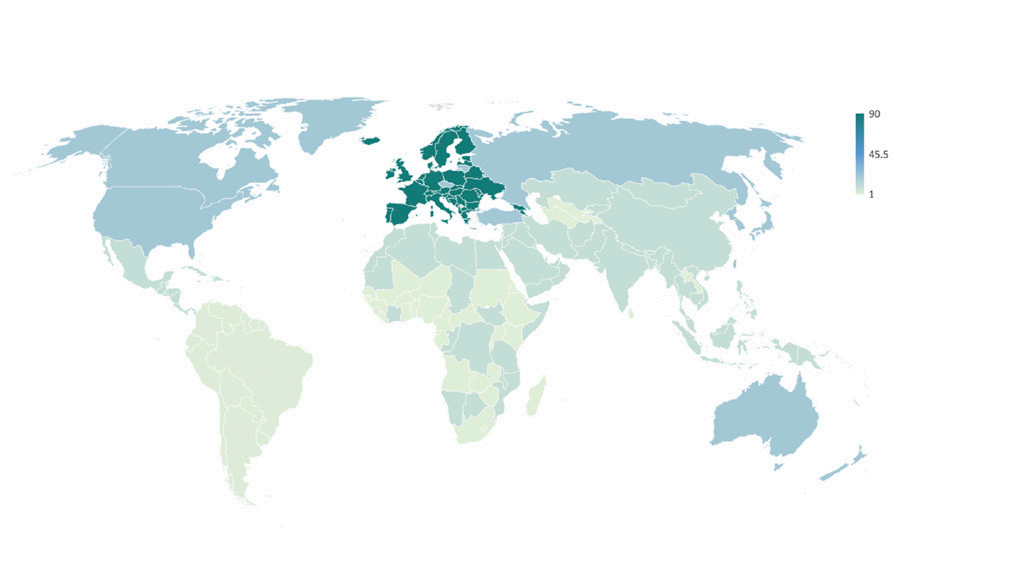
Total AMR reports by target locale* (2010s**−2020). *Excluding 112 worldwide reports. **Noting emergence of the Global South.

The Global North has and continues to dominate international AMR reporting. Although our results are biased towards Europe by including the EU as a transnational organisation but mostly excluding nation-level reports by individual governments, Northern countries are consistently over-represented both in terms of authorship and focus of AMR reports. Prior to the identified governance shift of international AMR policy (see above), most international reports focused on AMR as a problem of the Global North (see figure 5). As highlighted by Wernli *et al*,^14^ the Global South, while certainly acknowledged in earlier academic and national documents,^12^ only gained international traction as key to the AMR problem from around 2010 onwards (see figure 6). Since then, a growing number of international reports have focused on LMICs as part of the problem (mis-use/over-use) and as central to pan-national solutions. This new focus on the ‘South’ coincides with the discovery of prominent ‘Southern’ resistance factors like *ndm-1* (2008) that drive home global interconnectedness (what starts in Delhi, finds its way to London, New York and beyond) alongside the OH message (see below).^14 16^ It also coincides with IOs’ transition from AMR whistle-blowers to a new style of international health governance due to their political legitimacy to intervene in Southern countries (see discussion). Over the last decade, AMR has thus been reframed as a problem for the North but of the South.

### The ‘One Health’ pivot

**Figure 7:**
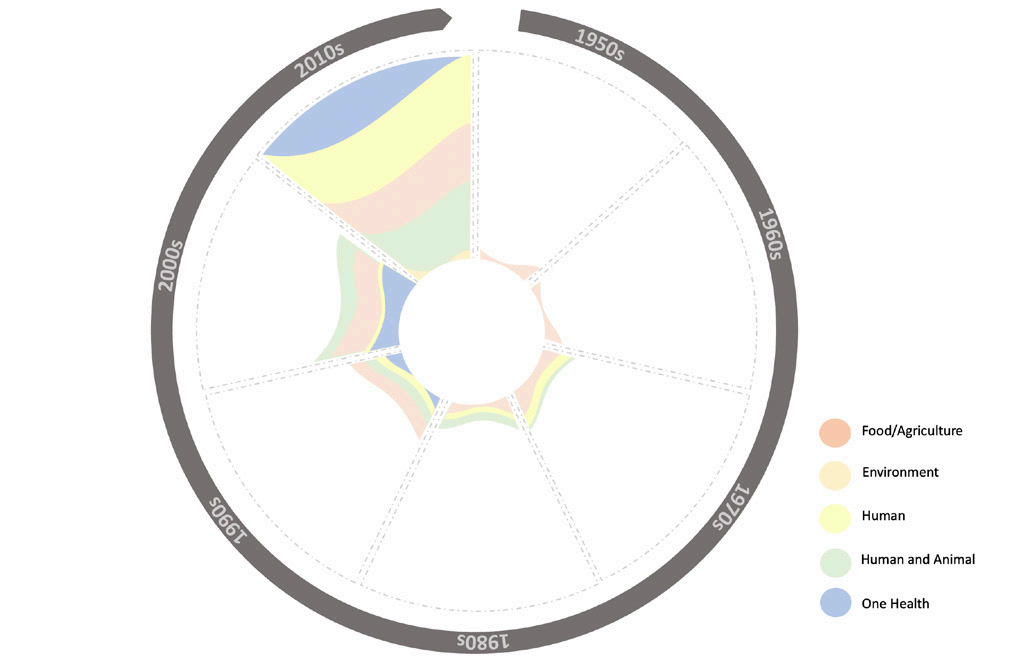
Orientation of AMR reports by decade (1950s–2010s).

Between 1945 and 2000, most international policy documents focused on agriculture and/or human antibiotic use and AMR selection (see figure 7). Since then, a new focus on OH has gradually entered AMR policy. The term OH, though circulated earlier (see discussion), was first captured in a joint 2008 report by WHO, FAO, OIE, UNICEF and the World Bank^24^ and is linked to pandemic preparedness and work in rural LMIC regions.^25–27^ Although use of OH terminology has grown exponentially, most international reports continue to focus on only two OH domains (humans and animals). The environmental domain is mostly conceived in terms of wildlife, while other components such as soil and water remain neglected. Of the 248 analysed documents, only two have exclusively and explicitly targeted the environment’s role in AMR selection via waste and pollution. The parallel emergence of OH and the ‘Global South’ in AMR rhetoric has been highlighted by other researchers^14 16^ and has been key to legitimising and unlocking finance for IOs’ shift to governance-based approaches.

### New framings, new interventions

**Figure 8:**
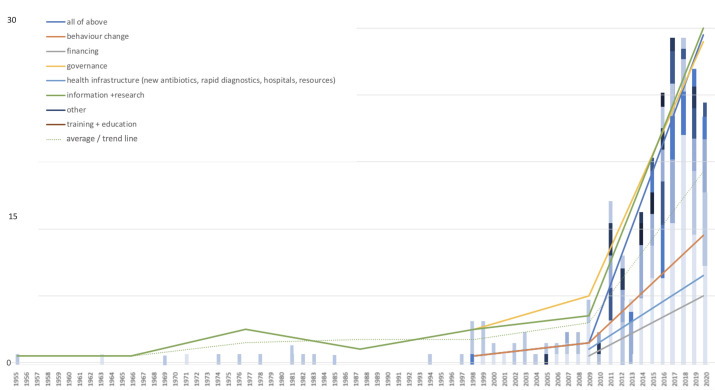
Intervention emphasis across total all AMR report types (1955–2020).

The interventions called for by the international AMR policy documents have also changed over time (see figure 8) and parallel the described geographical, OH and governance shifts of AMR framings.

### From ‘rational use’ to surveillance and stewardship

The rhetoric of reports targeting international antibiotic use, stewardship and AMR has closely followed wider moral economies. Mirroring postwar consumer movements,^3 28^ early documents focused on ensuring efficient or appropriate (‘rational’) antimicrobial use in distinct locales (hospitals, farms and community clinics). In contrast to this early focus on maximising antibiotic value, documents published from the mid-1990s onwards increasingly framed AMR as a problem of resource scarcity that was characterised by a lack of new drugs and should be tackled with conservationist strategies. The described shift in AMR discourse occurred unevenly but was in line with a broader reorientation of international debates towards issues of sustainability following the 1987 Brundtland Commission and 1992 Rio Climate Conference.^29^ Despite earlier Northern initiatives and the 1988 establishment of WHONet,^9 22 30^ it was not until the 2000s that integrated surveillance of antibiotic use and AMR emerged as prominent international policy objectives. Although various approaches to preserving antibiotic efficacy have been trialled since the 1940s,^2–4 11 12^ the term antimicrobial stewardship was first coined in 1996.^31^ Stewardship subsequently took years to become central to the internationally recognised two-pronged approach to addressing AMR (antibiotic innovation and preservation). Although definitions of stewardship remain amorphous, the term took hold as a sensitising concept around 2010.^32^ Reflecting the Global South lag of international policy, stewardship started with a focus on Northern contexts and was only gradually transferred to LMIC contexts. Aspects of first-wave ‘rational’ use discourse have nonetheless survived in the form of behaviourist interventions and hopes for precision medicine (eg, targeted diagnostics and treatments).

### The infrastructural turn

Initial international reports predominately called for more information and research. Governance and behaviour change at the level of the individual emerged as key aspects of international reporting during the 1990s. From around 2000 onwards, this was complemented by a focus on health infrastructures (hospital resources, farm biosecurity and integrated surveillance systems). The emerging ‘infrastructural turn’ paralleled calls for increased Northern financing to tackle AMR first at the national/regional and then—with the Southward expansion of stewardship discourse (see above)—at the international level. The post-2010 emphasis on the Global South has strengthened international calls for systems-level interventions in the form of improved water, sanitation and hygiene, infection prevention control and mass vaccination. In line with the described OH pivot, arguments for systems-level interventions are often framed using biosecurity terminology rather than in terms of collective structural responsibility. Resulting action plans remain primarily vertical and technology oriented and do little to address the horizontal socioeconomic factors underlying antibiotic consumption and threatening global ‘antibiotic infrastructures’.^33^ Meanwhile, the growing complexity and number of international AMR reports has not necessarily reflected mobilisation, but almost evidence of policy paralysis.

### More bugs, no drugs: antibiotic innovation

Antibiotic innovation is an outlier in this regard. Concerns about the ‘empty antibiotic pipeline’ were raised by industry and Northern policy reports in the early 1990s and began to feature in analysed reports from the early 2000s. The ‘empty pipeline’ narrative presents an attractive, tractable solution for AMR, a problem which was beginning to appear insolvable/ungovernable.^14 34^ Although it is difficult to quantify overall investment in stewardship, a disproportionate amount of the US$8.2 billion invested in AMR-related research and development projects since 2017 has targeted industry and innovation (almost US$3.4 billion) with most international investment focusing on human health (US$7352 million) rather than animals (US$956.2 million), the environment (US$234.8 million) or plants (US$85.6 million).^21^

### The top 10: AMR global experts

**Figure 9:**
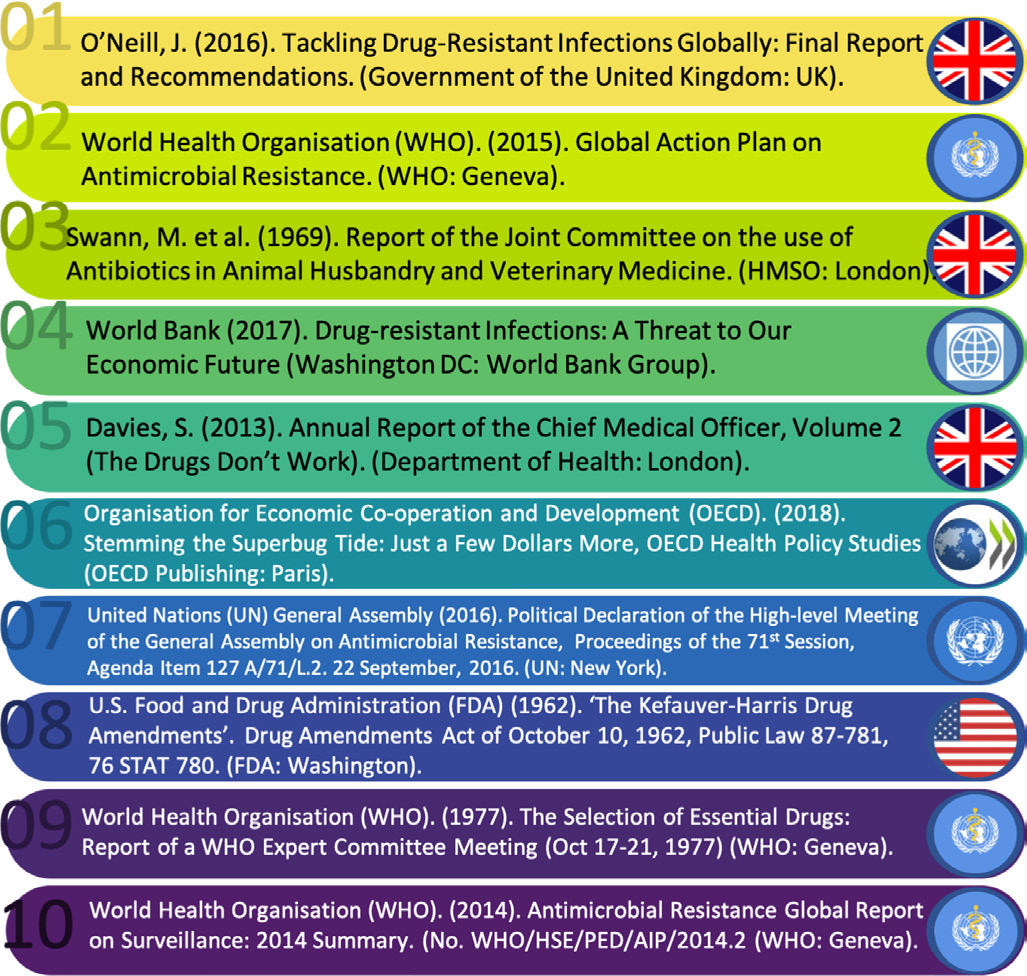
Top 10 AMR consensus reports as ranked by global experts.

The consultative process (part B) complemented and added value to the broader analysis of AMR reporting, through incorporating both informal but established knowledge of impact and significance. Due to their importance in opening the door for new approaches to safe-guarding antibiotic efficacy and conceptualising antibiotics as a global public good, the expert consultation led to the inclusion of documents like the 1977 WHO report on Essential Drugs (for the consensus and full coded list of reports, see online supplemental tables 2 and 3), which would not have been captured using the search parameters of part A. The consultation also highlighted the variable nature of perceived significance. For instance, our top ranked consensus report (see figure 9) *Tackling Drug-resistant Infections Globally: Final Report and Recommendations*,^35^ was ranked in the top 10 most influential reports by 87.5% of respondents, while the second top report (2015 WHO GAP) resulted in only 62.5% agreement. This reflects the important fact that reports gain different forms of traction across locales, contexts and disciplines, and with varying perceived impacts. However, what it also shows are patterns in broad significance, which map (although in non-uniform ways) across fields.

We are aware that the composition of our expert group may have impacted results. Of the 10 responding experts, three (30%) had significant expertise of antibiotic use and regulation in the African region, two (20%) in the European region, two (20%) in the US region, three (30%) in the Asian region and four (40%) of regulation at the IO level—with some respondents having expertise across multiple areas. None of our experts were specialists of the South American region and only two (20%) were scholars from the Global South. This means that our expert consultation may have missed policy documents targeting South America or reflecting South-to-South policy-making. We nonetheless believe that our identified top 10 of international reports highlight the salience of our broader content analysis (part A), illustrating no reports originating from or solely focusing on the Global South, a partial focus on OH (50%), and a dominance of the Global North in report authorship and focus (60% of reports, with the remainder WHO and UN) in perceived impact and significance.

## Discussion

Our analysis shows that international attempts to control AMR evolved in waves. Political attention waxed and waned as a result of competition from other policy areas and of different stewardship approaches running out of steam in the face of the biological, economic, social and political complexities of AMR. The framing of AMR risks also changed over time and was linked to varying degrees of optimism or pessimism about the ability of antibiotic development to ‘keep up’ with AMR as well as with broader changes in political discourse relating to consumerism and sustainability.^2–4^

Awareness of AMR as a clinical problem dates back to the interwar period and national policies for ‘rational’ antibiotic use in human and animal medicine emerged during the 1940s. At the international level, AMR first emerged as a policy challenge in reaction to concerns about expanding agricultural antibiotic use and the global spread of resistant *Staphylococcus aureus* 80/81.^3 4 11 12^ While early reports focused on antibiotic residues and infection control in human medicine, 1960s concerns about horizontal gene transfer led to international risk assessments of low-dosed antibiotic use on farms but no integrated action plans. More sustained international action arose in response to the European Economic Community’s (EEC) 1970 precautionary antibiotic feed restrictions, ultimately abortive US ban initiatives, and regional outbreaks of multiple drug resistant (MDR) gram-negative organisms.^2–4 11–13 36 37^ Between the mid-1970s and early 1980s, WHO expert committees reviewed national antibiotic policies primarily with regard to agriculture and the disputed impact of antibiotic feed bans on AMR. Although campaigners like Stuart Levy tried to reframe AMR as a global challenge, the international community mostly targeted antibiotic usage and AMR in the Global North and West of the Iron Curtain.^2–4 12^ Amidst a wider reorganisation of international health and a relative weakening of WHO influence during the late 1980s,^38^ international efforts to address AMR, however, diminished.

AMR re-emerged as a target of international policy during the mid-1990s in response to Northern warnings about the biosecurity threats posed by emerging infectious diseases, outbreaks of MDR pathogens like vancomycin resistant enterococci and stalling antibiotic innovation. Antibiotic use in agriculture continued to be a strong focus of international reports.^2 4 11–13 39^ While earlier international reports had often framed AMR as a challenge of rational use, this second phase of reporting reframed AMR as a problem of scarcity and sustainability. Published in parallel to EU antibiotic feed bans and the creation of integrated Northern AMR surveillance, international expert reports endorsed therapeutic reservations of medically relevant drugs and called for expanded monitoring.^4^ AMR’s environmental dimensions were rarely discussed and most reports remained focused on the Global North. Despite a parallel surge of MDR in the Global South,^37 40^ international initiatives targeting LMICs remained limited and IOs primarily acted as reviewers and whistle-blowers. Following an ultimately fruitless attempt by UN IOs to galvanise coordinated action on 11 September 2001,^23^ international attention for AMR dissipated as a result of concerns about pandemic preparedness and bioterrorism.^41 42^

The re-emergence of AMR as an international concern between 2010 and 2015 occurred against the backdrop of a reordered landscape of global health politics. Since 2003, numerous pandemic crises (SARS, avian Influenza, H1N1, H5N1) had given rise to a new system of international health governance focused on biosecurity. Concerns about (re-)emerging infections at the intersection of human and animal health strengthened UN IOs’ role as the only institutional actors capable of staging politically legitimate interventions in sovereign (Southern) territories. Enhanced sentinel surveillance and the rapid deployment of *prêt-à-porter* policy instruments like travel restrictions in response to identified threats were at the heart of this interventionist mode of governance, which was consolidated by the passage of the reformed International Health Regulations (IHR) in 2005.^41 43–46^ Framing global health in terms of biosecurity did not create equals. As the example of avian influenza shows, lack of domestic resources and expertise meant that LMICs like Vietnam often had little choice other than to accept IO policy instruments if they wanted to remain part of the international community of trade and nations.^47^

The described changes also affected AMR politics. Amidst a rapid increase in the number of antibiotic reports, IOs’ role transitioned from raising awareness and reviewing data to managing the emerging physical and legal frameworks underpinning global antibiotic governance. Regulatory tools such as action plans and surveillance systems as well as broader instruments like the new IHR^46^ or OIE’s Terrestrial Animal Health Code (OIE)^48^ helped institutionalise the new system of IO-led governance. Geopolitical interests were embedded in this governance system. In the case of middle powers like the UK, targeted engagement with AMR offered an attractive way to gain prestige by showing ‘global leadership’ (see below) and exert soft power in LMICs. As evidenced by our expert report ranking (figure 9), it is no coincidence that two British-authored reports from the last decade are in the top five or that British experts and donor initiatives play a very prominent role in international antibiotic politics.

The transition towards AMR governance was accompanied by an increasing focus on OH and the Global South. The parallel rise of both terms in international reporting is highly significant and reflected a growing realisation of the complexities of AMR as a global problem that was not amenable to interventions targeting high-income countries (HICs) alone.^39^ Similar to the rhetoric surrounding bioterrorism and pandemic responses,^42 49^ NGOs and budget-constrained UN IOs made investing in global stewardship meaningful to Northern donors by mobilising historical tropes of ‘dangerous’ tropical environments^49 50^ as a source of territorial and economic risk. Profiting from rising funding for Global Health-oriented research, academics also played an important role in driving the Southern reorientation of Northern AMR attention.

The 2008 detection of transferable *New Delhi metallo-beta lactamase 1* (*ndm-1*) in *Klebsiella pneumoniae* is emblematic of this reframing of AMR as a Southern threat. The gene enabling enzyme production had first been identified in a carbapenem resistant strain isolated in Sweden.^51 52^ The new plasmid and enzyme were named after its detection in a patient who had recently been treated in New Delhi and international researchers subsequently concluded that the strain had originated on the Indian subcontinent. Prominent Indian politicians subsequently complained about geographic stigmatisation and potential impacts on medical tourism but also blocked further research.^53 54^ Although *Lancet* editor Richard Horton apologised for reprinting the geographic name tag and numerous other transferable carbapenemases had been detected across the world since the early 1990s,^55 56^ *ndm-1* quickly became a poster child for the ‘Southern’ reconceptualisation of AMR threats. International policy reports, academic papers and media reports soon used it as a key reference point in arguing for increased Northern investment in global—and primarily LMIC—stewardship.^57–59^ The planetary scale of AMR threats and need for corresponding global action was often expressed using environmental metaphors. In her report for 2011, UK Chief Medical Officer—and current UK AMR Envoy—Sally Davies referenced the import of *ndm-1* alongside climate change, zoonotic viruses and coastal flooding to galvanise British action on AMR.^60^

The international reframing of AMR as a ‘Southern’ risk was paralleled by the adoption of OH rhetoric. Tracing its roots back to Calvin Schwabe’s 1960s One Medicine philosophy and colonial era tropical medicine, OH had emerged as a distinct discipline focusing on human, animal and environmental health around 2000. By 2008, the WHO, FAO and OIE had adopted OH approaches in response to zoonotic threats.^26 27 61 62^ In the case of AMR, OH’s holistic focus seemed ideally suited to developing cross-domain stewardship programmes. OH’s focus on interconnected disease environments and attractive ‘One World, One Health’ rhetoric suited the Global Health philosophies of major funders and legitimised the expansion of UN IOs’ role in coordinating global AMR initiatives. By reinforcing tropes of dangerous Southern disease- and AMR-scapes,^61^ OH imagery, however, also led to a relative deterritorialisation of AMR politics, which justified exercising pressure for AMR mitigation in Southern countries.

Quantifying economic and health threats was an important part of the described policy shift towards international AMR governance and new Global South and OH rhetoric. In 2014, British Prime Minister David Cameron commissioned an independent economic review of AMR by former Goldman Sachs economist Terence James O’Neill. Underlining the role of soft power interests within international AMR governance, the initiative was explicitly framed as a way for ‘Britain to lead the way, using its international leadership and world-class pharmaceutical sector (…) to battle against antimicrobial resistant infections and bring new drugs to the world market’.^63^ The final 2016 report proposed a mix of behavioural, structural and biomedical interventions and estimated that effective global action over 10 years would cost up to US$40 billion. This compared with up to 10 million annual AMR-related deaths by 2050 resulting in US$100 trillion costs if no action was taken. BRICS (Brazil, Russia, India, China, South Africa)—a term O’Neill had coined in 2001^64^—were identified as areas of particular concern due to rising drug usage and AMR.^35^ The 2015 detection of the transferable mobilised colistin resistance (*mcr-1*) gene in Chinese pigs seemingly confirmed concerns and prompted international pressure on major users like China and Brazil to outlaw polymyxin feed additives.^65–67^ Subsequent analysis, however, showed that *mcr-1* was detectable in strain collections from more than 30 countries and was already circulating in China in the mid-1980s.^8 68^

The *mcr-1* episode and O’Neill report coincided with a peak of international action. On 26 May 2015, a tripartite initiative by WHO, OIE and FAO led to the passage of a global action plan on AMR by the World Health Assembly.^1^ HICs and LMICs agreed on an ambitious programme of national action plans (NAPs) against AMR, a WHO-led Global Antibiotic Surveillance System^69^ and improved OIE usage surveillance.^70 71^ Couched as a global OH challenge, AMR appeared on the agendas of the G7,^72^ OECD,^73^ G20^74^ and UN^75^ with HIC governments and donors committing substantial funds to developing diagnostics and antibiotics, to strengthening surveillance in LMICs and to related programmes involving strengthening water, sanitation and hygiene systems and vaccination. At the regional level, it also galvanised action like the creation of dedicated AMR arms and action plans by the African Union’s newly founded Africa Centres for Disease Control and Prevention.^76^ Ensuing NAPs, surveillance reports and funding strategies led to a surge of international publications and unlocked new political and financial resources for the IOs coordinating global action.

How long this surge of international attention for AMR will last and what its impacts will be is uncertain. Arguably, AMR awareness is already past its prime. Even before the reorientation of political agendas towards COVID-19, the annual number of international reports dedicated to AMR had peaked in 2017. This was despite an ongoing rise of global antimicrobial usage and AMR, an international outbreak of extensively drug resistant typhoid, stuttering antibiotic innovation and an ongoing neglect of environmental health within OH frameworks.^77^ As critical reviews of many NAPs indicate,^78–80^ it remains unclear what impact the extension of international AMR governance has had at the national and regional levels. The disconnect between the universal language and Northern authorship of most international antibiotic reports highlighted in our analysis is bound to impact action on the ground. There is thus an urgent need for case studies involving LMIC archives and stakeholders to help us better understand how legitimacy for AMR governance is—or fails to be—coproduced by interactions between local and international policy-makers.

Meanwhile, there are also signs of a growing divergence of problem analysis and political action at the international level. Recent reports by the World Bank,^81^ UN IACG^75^ and academics^8^ have responded to the described complexities and deterritorialisation of AMR politics by mixing calls for structural and behavioural interventions. However, most international investment seems to continue to focus on technical solutions like pharmaceutical innovation and market-based reform.^21 82 83^ While rising investment in AMR mitigation is undoubtedly welcome, conceiving of stewardship as a challenge that can be primarily overcome via novel technologies, vaccines or focused investment in microbial surveillance does not necessarily work in territories with robust political, economic, health and agricultural infrastructures and can easily overburden already weakly (formally) governed environments.^8^

Despite AMR’s reappearance on the agendas of the 2021 G7 and a high-level UN interactive virtual meeting,^84 85^ the seeming fall of international attention and cuts to AMR focused aid and budgets by major international donors like the UK should worry us.^20 21^ On the one hand, it marks a prolongation of a well-established pattern. Since 2016, other issues like the climate emergency, nationalist politics and COVID-19 have likely distracted from international AMR initiatives—despite the pandemic-related surge of international health expenditure. On the other hand, waning international attention also points to the underlying intractability of AMR as a policy problem. Seen from the vantage point of 2021, the historical dilemma of international AMR policy has been that it is both too discreet and too big for its own good. When framed as a problem of drugs failing, AMR repeatedly attracted ‘quick fix’^86^ solutions like partial restrictions, narrow behavioural interventions and prioritisation of innovation over stewardship, which often failed in the face of complex interconnected AMR ecologies and infectious disease challenges in both LMIC and HIC contexts.^8 87 88^ However, when analysed in its full complexity, tackling AMR quickly becomes a challenge of tackling everything everywhere and well-intentioned policy initiatives stutter and stall.

Greater awareness of the ways in which AMR is framed as an object of global attention and subject of international intervention seems key to breaking the described cycle of optimism and lethargy. Although the post-2010 wave of attention has led to novel and more complex policy solutions as well as lasting stewardship alliances, it should not make us blind to the geopolitics, Northern biases and (post)colonial tropes that are couched in the deterritorialised OH rhetoric of recent reports and action plans. It is clear that AMR will not be tackled by national politics and—in the absence of other credible actors—IOs’ increased involvement is to be welcomed. However, by continuing to frame AMR according to the biosecurity preoccupations of Northern donors, IOs and academics alike risk ignoring Southern perspectives, perpetuating a history of distrust and jeopardising LMIC buy-in and prioritising regional over planetary solutions. In the long-term, effective collective action on planetary challenges will only result from international partners working together as equals.

## Data Availability

All data relevant to the study are included in the article or uploaded as supplementary information.
