# Supplementary material for: Waves of attention: patterns and themes of international antimicrobial resistance reports, 1945–2020
Source: BMJ Glob Health. 2021 Nov 5;6(11):e006909. doi: 10.1136/bmjgh-2021-006909 (PMC8573652; doi:10.1136/bmjgh-2021-006909)
Supplement: Supplementary data [file bmjgh-2021-006909supp001.pdf]

BMJ Publishing Group Limited (BMJ) disclaims all liability and responsibility arising from any reliance placed on this supplemental material which has been supplied by the author(s)

Supplementary Table One: Coding Categories

**Type of report**	**Publisher of report**	**Orientation**	**Target audience**	**Target locale**	**Stage of policy/report**	**Aim of policy report**	**Intervention called for**
action plan	government	human	academics	Africa	contemplative	access	financing
scientific report	NGO	animal	general population	Americas	implementation/action	Conservation	behaviour change
surveillance report	private	plant	government	Asia	maintenance	containment	governance
stakeholder engagement		environment	industry	Europe	evaluating	infection prevention	training + education
legislation		food	healthcare sector	Oceania	other	innovation	health infrastructure
policy framework		one health	farmers/vets	Global north		surveillance	information +research
meeting report		human & animal	Other	Global south		Other	other
		Agriculture		World wide		All of above	
