# Supplementary material for: Waves of attention: patterns and themes of international antimicrobial resistance reports, 1945–2020
Source: BMJ Glob Health. 2021 Nov 5;6(11):e006909. doi: 10.1136/bmjgh-2021-006909 (PMC8573652; doi:10.1136/bmjgh-2021-006909)
Supplement: Supplementary data [file bmjgh-2021-006909supp002.pdf]

Supplementary Table Two: Coded Reports

Year	Title of Report
1955	NAS. *Proceedings First International Conference on the Use of Antibiotics in Agriculture* (Washington: National Academy of Sciences - National Research Council, 1955).
1961	WHO. *Standardization of methods for conducting microbic sensitivity tests: second report of the Expert Committee on Antibiotics [meeting held in Geneva from 11 to 16 July 1960]* (Geneva: WHO, 1961).
1963	WHO. *The Public Health Aspects of the Use of Antibiotics in Food and Feedstuffs, World Health Organization* [Technical Report Series 260] (Geneva: WHO, 1963).
1969	NAS/NRC: *The use of drugs in animal feeds. Proceedings of a symposium 1969* (Washington: NAS/NRC, 1969).
1970	EEC. Council Directive 70/524/EEC concerning additives in feedingstuffs. *Official Journal of the European Union.* L 270, 14 December 1970, pp. 1-17.
1971	Ericsson, Hans M., and John C. Sherris. Antibiotic Sensitivity Testing. Report of an International Collaborative Study. Vol. Suppl. 217, *Acta Pathologica et Microbiologica Scandinavica*. Copenhagen: Munksgaard, 1971.
1974	WHO. *The Public Health Aspects of Antibiotics in Foodstuffs. Report on a Working Group, Bremen* (1-5 October 1973) (Geneva: WHO, 1974).
1976	WHO. *Public Health Aspects of Antibiotic-resistant Bacteria in the environment report on a consultation meeting, Brussels, 9-12 December 1975* (Geneva: WHO, 1976).
1977	WHO. *The Selection of Essential Drugs* (Geneva: WHO, 1977).
1978	WHO. *Surveillance for the prevention and control of health hazards due to antibiotic-resistant enterobacteria: report of a WHO meeting* (Geneva: WHO, 1978).
1981	WHO. *Antimicrobial resistance report of scientific working group Geneva, 23-27, Nov* (Geneva: WHO, 1981).
1981	Levy, S., Clowes, R. and Koenig, E. (conveners). *Statement Regarding Worldwide Antibiotic Misuse. Molecular Biology, Pathogenicity, and Ecology of Bacterial Plasmids Conference* (Santo Domingo, 1981)
1982	WHO. *Surveillance of antimicrobial resistance repost of a consultation* (Geneva: WHO, 1982).
1983	WHO. *Control of antibiotic-resistant bacteria: Memorandum from a WHO Meeting* (Geneva: WHO, 1983).
1985	Burke, John P, Summary report of worldwide antibiotic resistance: international task forces on antibiotic use, *Reviews of Infectious Diseases* 7/4 (1985).
1994	WHO. *WHO scientific working group on monitoring and management of bacterial resistance to antimicrobial agents* (Geneva: WHO, 1994).
1997	WHO. *The Medical Impact of Antimicrobial Use in Food Animals. Report of a WHO Meeting. Berlin, Germany, 13-17 October 1997* (Geneva: WHO, 1997).
1998	*Report from the Invitational European Union Conference: 'The Microbial Threat: The Copenhagen Recommendation* (Copenhagen: Danish Ministry of Health and Ministry of Food Agriculture and Fisheries, September 1998).

1998	WHO. *World Health Assembly (51st), Emerging and other communicable diseases: antimicrobial resistance. WHA51.17, 1998, agenda item 21.3.* (Geneva: WHO, 1998).
1998	WHO. *Use of Quinolones in Food Animals and Potential Impact on Human Health. Report of a WHO Meeting, Geneva, Switzerland, 2-5 June 1998* (Geneva: WHO, 1998).
1998	WHO. *Antimicrobial Resistance Monitoring: Information Exchange and Opportunities for Collaboration. Report of the Second Joint Who/Ifpma Meeting, Geneva 2-3 April 1998* (Geneva: WHO, 1998).
1999	*Opinion of the Scientific Steering Committee on antimicrobial resistance. European Commission, Directorate- General XXIV* (Brussels: SSC for EC, 1999).
1999	OIE. *The use of antibiotics in animals - Ensuring the protection of public health*. Summary and Recommendations from the European Scientific Conference 24-26 March, Paris. (Paris: OIE, 1999).
1999	European Agency for the Evaluation of Medicinal Products, *Antibiotic resistance in the European Union associated with therapeutic use of veterinary medicines. Report and qualitative risk assessment. Committee for Veterinary Medicinal Products. July 14* (London: EMA, 1999).
2000	EMA. *A risk management strategic plan for controlling antimicrobial resistance through the authorisation of veterinary medicines. Recommendations consequent to the report and qualitative risk assessment of the CVMP* (London: EMA, 2000).
2000	WHO/ FAO/OIE. *WHO Global Principles for the Containment of Antimicrobial Resistance in Animals Intended for Food. Report of a WHO Consultation with the participation of the Food and Agriculture Organization of the United Nations and the Office International des Epizooties*. Geneva, Switzerland. June 5-9 (WHO: Geneva, 2000).
2001	WHO. *WHO global strategy for containment of antimicrobial resistance* (WHO: Geneva, 2001).
2002	WHO. *Monitoring antimicrobial usage in food animals for the protection of human health Report of a WHO consultation Oslo, Norway 10-13 September 2001* (Geneva: WHO, 2002).
2002	EC, *Council recommendation of 15 November 2001 on the prudent use of antimicrobial agents in human medicine* (Brussels: European Commission, 2002).
2003	European Parliament. *Regulation (EC) No 1831/2003 of the European Parliament and the Council of 22 September 2003 on additives for use in animal nutrition. Official Journal of the European Union, L268/29, 18.10.2003* (Strasbourg: EP, 2003).
2003	OIE, *OIE International Standards on Antimicrobial Resistance. Paris: OIE. Based on 2001 OIE Headquarters meeting Paris* (Paris: OIE, 2003).
2003	FAO/WHO/OIE. *Joint FAO/WHO/OIE Expert Workshop on Non-Human Antimicrobial Usage and Antimicrobial Resistance: Scientific Assessment. Geneva, Switzerland, 1-5 December 2003* (Geneva: WHO, 2003).
2003	WHO, *Impacts of antimicrobial growth promoter termination in Denmark : the WHO international review panel' s evaluation of the termination of the use of antimicrobial growth promoters in Denmark : Foulum, Denmark 6-9 November 2002* (Geneva: WHO, 2003).

2004	FAO/WHO/OIE. 2004. *Second Joint FAO/WHO/OIE Expert Workshop on Non-Human Antimicrobial Usage and Antimicrobial Resistance: Management Options. Oslo, Norway, 15-18 March 2004* (Geneva: WHO, 2004).
2005	FAO/WHO/Codex Alimentarius. *Code of Practice to minimize and contain antimicrobial resistance* (2005).
2005	WHO. *Critically important antibacterial agents for human medicine for risk management strategies of non-human use. Report of a WHO working group consultation, Canberra, Australia, 15-18 February 2005* (Geneva: WHO, 2005).
2006	EMA. *CVMP Strategy on Antimicrobials 2006-2010 and status report on activities on antimicrobials* (London: EMA, 2006).
2006	FAO/WHO/OIE. *Report of a Joint FAO/WHO/OIE Expert Consultation on Antimicrobial Use in Aquaculture and Antimicrobial Resistance. Seoul, Republic of Korea, 13-16 June 2006* (Geneva: WHO, 2006).
2007	OIE. *OIE list of antimicrobials of veterinary importance. November 2007* (Paris: OIE, 2007).
2007	WHO. *Critically important antimicrobials for human medicine: categorization for the development of risk management strategies to contain antimicrobial resistance due to non-human antimicrobial use. Report of the second WHO Expert Meeting, Copenhagen, Denmark, 29-31 May 2007* (Geneva: WHO, 2007).
2007	EFSA. 'Report of the Task Force of Zoonoses Data Collection including a proposal for harmonized monitoring scheme of antimicrobial resistance in Salmonella in fowl (Gallus gallus), turkeys, and pigs and Campylobacter jejuni and C. coli in broilers', *EFSA Journal* (2007).
2008	FAO/WHO/OIE. *Joint FAO/WHO/OIE Expert Meeting on Critically Important Antimicrobials. Report of the FAO/WHO/OIE Expert Meeting. FAO, Rome, 26-30 November 2007* (Rome: FAO, 2008).
2008	EC, *Council Conclusions on Antimicrobial Resistance* (Brussels: Council of European Union, 2008).
2008	WHO, FAO, OIE, UNICEF, UNSIC, and World Bank. *Contributing to One World, One Health – A Strategic Framework for Reducing Risks of Infectious Diseases at the Animal-Human-Ecosystems Interface.*
2009	EC. *Council Conclusions of 1 December 2009 concerning innovative incentives for effective antibiotics* (Brussels: European Commission, 2009).
2009	ECDC, EMEA. *The bacterial challenge: time to react, ECDC/EMEA joint technical report, European Centre for Disease Prevention and Control, Stockholm* (Stockholm: ECDC 2009).
2009	ECDC/EFSA/EMA. 'Joint Scientific Report of ECDC, EFSA and EMEA on Methicillin Resistant Staphylococcus aureus (MRSA) in Livestock, Companion Animals and Food', *EFSA Journal*, 7 (6) (2009).
2009	ECDC/EFSA/EMA. 'Joint Opinion on antimicrobial resistance (AMR) focused on zoonotic infections'. *EFSA Journal*, 7 (2009).
2009	EFSA. *Assessment of the Public Health significance of methicillin resistant Staphylococcus aureus (MRSA) in animals and foods Scientific Opinion of the Panel on Biological Hazards(Question No EFSA-Q-2008-300) Adopted on 5 March 2009* (Parma: EFSA, 2009).

2009	EMEA/HMA. *Joint Heads of Medicines Agencies (HMA)/EMEA Meeting on Promoting Prudent and Responsible Use of Antimicrobials in Veterinary Medicine Marienbad, Czech Republic 19 – 20 May 2009*.
2010	FAO, OIE, WHO. *The FAO-OIE-WHO collaboration – tripartite concept note, FAO, OIE and WHO* (WHO/OIE/FAO, 2010).
2010	ECDC. *AMR surveillance in Europe in 2009* (Stockholm: ECDC, 2010).
2010	EC. *CVO conclusions on AMR* (Brussels: Council of European Union, 2010).
2011	EC. Action plan against the rising threats from AMR Communication from the Commission to the European Parliament and the Council (Brussels: Council of European Union, 2011).
2011	*European Parliament resolution of 27 October 2011 on the public health threat of antimicrobial resistance* (2013/C131 E/14).
2011	*European Parliament resolution of 12 May 2011 on antibiotic resistance* (P7_TA(2011)0238.
2011	FAO Codex. *Guidelines for risk analysis of foodborne antimicrobial resistance* (FAO/ Codes, 2011).
2011	*EU US Joint workshop. Challenges and Solutions in the Development of New Diagnostic Tests to Combat Antimicrobial Resistance* (2011).
2011	TATFAR. *Transatlantic Taskforce on Antimicrobial Resistance. Recommendations for future collaboration between the US and EU 2011* (2011).
2011	ECDC. *AMR surveillance in Europe in 2010* (EARS-Net report, 2011).
2011	EFSA /ECDC. 'The European Union Summary Report on antimicrobial resistance in zoonotic and indicator bacteria from human, animals and food in the European Union in 2009', *EFSA Journal* 9:2154 (2011).
2011	EMA. *CVMP strategy on antimicrobials 2011-2015* (London: EMA, 2011).
2011	EMA. *Trends in the sales of veterinary antimicrobial agents in nine European countries 2005-2009* (first ESVAC report) (London: EMA, 2011).
2011	WHO Euro Region. *Tackling antibiotic resistance from a food safety perspective in Europe* (Copenhagen: WHO Regional Office for Europe, 2011).
2011	Leung, Emily et al. World Health Day, policy package to combat AMR with critical actions by all stakeholders, *Bull World Health Organ* 89/5 (2011), 390-392.
2011	WHO. *Critically Important Antimicrobials for Human Medicine: 2nd revision* (Geneva: WHO, 2011).
2011	WHO. *European Strategic action plan on AMR* (Geneva: WHO, 2011).
2011	HMA. *HMA-V Action plan on antimicrobial issues Updated Version for publication (7 November 2011)* (Dublin: HMA, 2011).
2011	European Commission. *Communication from the Commission to the European Parliament and the Council. Action plan against the rising threats from antimicrobial resistance*. COM(2011) 748 (Brussels: European Commission, 2011).
2011	World Health Organisation (WHO) South-East Asia Region. *Jaipur declaration on antimicrobial resistance. WHO Regional Office for South-East Asia: Jaipur, India* (WHO SEARO: New Delhi, 2011).

2012	*European Parliament resolution of 11 December 2012 on the Microbial Challenge — Rising threats from Antimicrobial Resistance (2012/2041(INI)).*
2012	*European Parliament Report on the Microbial Challenge – Rising threats from Antimicrobial Resistance* (2012).
2012	Council of the European Union. *Council conclusions of 22 June 2012 on the impact of antimicrobial resistance in the human health sector and in the veterinary sector — a 'One Health' perspective.*
2012	FAO. *Improving biosecurity through prudent and responsible use of veterinary medicines in aquatic food production. Fisheries and aquaculture technical paper* (FAO: Rome, 2012).
2012	OIE. 'Antimicrobial resistance in animal and public health', *Scientific and Technical Review* 31/1 (2012), 15-21.
2012	WHO. *The Evolving Threat of Antimicrobial Resistance: Options for Action* (Geneva: WHO, 2012).
2012	WHO. *Critically Important Antimicrobials for Human Medicine: 3rd revision* (Geneva: WHO, 2012).
2012	ECDC. *AMR surveillance in Europe in 2011 (EARS-Net report)* (Stockholm: ECDC, 2012).
2012	EFSA /ECDC. 'The European Union Summary Report on antimicrobial resistance in zoonotic and indicator bacteria from humans, animals and food in 2010', *EFSA Journal* 10:2598 (2012).
2012	EMA. *Sales of veterinary antimicrobial agents in 19 EU/EEA countries in 2010 (second ESVAC report)* (London: EMA, 2012).
2013	European Commission. *Antimicrobial Resistance, Special Eurobarometer 407 Report* (Brussels: European Commission, 2013).
2013	EFSA/ECDC. 'The European Union Summary Report on antimicrobial resistance in zoonotic and indicator bacteria from humans, animals and food in 2011', *EFSA Journal* 11/5 (2013), 3196.
2013	ECDC. *AMR surveillance in Europe in 2012 (EARS-Net report)* (Stockholm: ECDC, 2013).
2013	ECDC. *Surveillance of antimicrobial consumption in Europe in 2010* (ESAC report) (Stockholm: ECDC, 2013).
2013	EMA. *Sales of veterinary antimicrobial agents in 25 EU/EEA countries in 2011 (ESVAC report)* (London: EMA, 2013).
2013	FAO. Addressing antimicrobial resistance, *FAO Highlights*, July 2013.
2014	*67th World Health Assembly resolution WHA67.25 for global action plan in May 2014.*
2014	APEC. *APEC Guideline to Tackle Antimicrobial Resistance in the Asia-Pacific Region, APEC* (Seoul: APEC, 2014).
2014	Council of the European Union. 'Council conclusions of 1 December 2014 on patient safety and quality of care, including the prevention and control of healthcare associated infections and antimicrobial resistance', *Official Journal of the European Union* C438/7 (2014).
2014	TATFAR. *TATFAR : Progress report Recommendations for future collaboration between the US and EU* (2014).

2014	EMA. *Answers to the requests for scientific advice on the impact on public health and animal health of the use of antibiotics in animals* (London: EMA, 2014).
2014	*EIP-AGRI Focus Group Reducing antibiotic use in pig farming* (European Commission, 2014).
2014	ECDC. *AMR surveillance in Europe in 2013 (EARS-Net report)* (Stockholm: ECDC, 2014).
2014	ECDC. *Surveillance of antimicrobial consumption in Europe in 2012 (ESAC report)* (Stockholm: ECDC, 2014).
2014	ECDC. *Surveillance of antimicrobial consumption in Europe in 2011 (ESAC report)* (Stockholm: ECDC, 2014).
2014	EMA. *Sales of veterinary antimicrobial agents in 26 EU/EEA countries in 2012 (ESVAC report)* (London: EMA, 2014).
2014	WHO. *Antimicrobial resistance: global report on surveillance* (Geneva: WHO, 2014).
2014	WHO. *Briefing Note Antimicrobial Resistance: An Emerging Water, Sanitation and Hygiene Issue* (Geneva: WHO, 2014).
2014	FAO. *Review of the Literature on Antimicrobial Resistance in Zoonotic Bacteria from Livestock in East, South and Southeast Asia* (Rome: FAO, 2014).
2015	TATFAR. *Report on the modified Delphi process for common structure and process indicators for hospital antimicrobial stewardship programs* (2015).
2015	G7. *Declaration of the G7 Health Ministers Berlin Declaration on Antimicrobial Resistance Global Union for Antibiotics Research and Development* (GUARD) – 8 - 9 October 2015 in Berlin (Berlin, 2015).
2015	G7. *Leaders' Declaration G7 Summit 7-8 June 2015*. G7 Germany, Elmau (Elmau, 2015).
2015	*European Parliament Resolution of 19 May 2015 on safer healthcare in Europe: improving patient safety and fighting antimicrobial resistance* (P8_TA(2015)0197).
2015	EC. 'Guidelines for the prudent use of antimicrobials in veterinary medicine', *Official Journal of the European Union* C 299/04 (2015).
2015	EC. *Progress report on the action plan against the rising threats from AMR* (SWD (2015) 59) (Brussels: European Commission, 2015).
2015	ECDC. *Annual epidemiological report 2014. Antimicrobial resistance and healthcare-associated infections* [2012 data] (Stockholm: ECDC, 2015).
2015	ECDC. *AMR surveillance in Europe in 2014 (EARS-Net report)* (Stockholm: ECDC, 2015).
2015	EMA. *Sales of veterinary antimicrobial agents in 26 EU/EEA countries in 2013 Fifth ESVAC report* (London: EMA, 2015).
2015	ECDC/EFSA/EMA. 'First joint report on the integrated analysis of the consumption of antimicrobial agents and occurrence of antimicrobial resistance in bacteria from humans and food-producing animals', *EFSA Journal* 13/114 (2015) (Joint Interagency Antimicrobial Consumption and Resistance Analysis (JIACRA).
2015	OECD. *Policy brief AMR in G7 countries* (Paris: OECD, 2015).

2015	OECD. *Antimicrobial Resistance in G7 Countries And Beyond: Economic Issues, Policies and Options for Action* (Paris: OECD, 2015).
2015	WHO. *Global Action Plan on Antimicrobial Resistance* (Geneva: WHO, 2015) (developed together with FAO and OIE).
2015	WHO. *Worldwide country situation analysis: response to antimicrobial resistance* (Geneva: WHO, 2015).
2015	WHO. *Antibiotic Resistance. A multi-country public awareness survey* (Geneva: WHO, 2015).
2015	WHO. *Global Antimicrobial Resistance Surveillance System: Manual for Early Implementation* (Geneva: WHO, 2015).
2015	FAO. *Codex texts on foodborne antimicrobial resistance* (Rome: FAO, 2015).
2015	FAO. *Status Report on Antimicrobial Resistance* (Rome: FAO, 2015).
2015	FAO. 'FAO's work on antimicrobial resistance', *Information Note* no. 4 CL 153/3 Adjustments to the Programme of Work and Budget 2016-2017. (Rome: FAO, 2015).
2016	WHO. *Critically Important Antimicrobials for Human Medicine: 4th Revision* (Geneva: WHO, 2016).
2016	UN. *Political Declaration of the UN General Assembly on Antimicrobial Resistance (71st United Nations General Assembly Resolution 70/183) to hold high-level meeting on AMR* (New York: UN, 2016).
2016	Council of the European Union. 'Council conclusions on the next steps under a One Health approach to combat antimicrobial resistance', *Official Journal of the European Union* C 259/26 (2016).
2016	EMA /EFSA. 'Joint Scientific Opinion on measures to reduce the need to use antimicrobial agents in animal husbandry in the European Union, and the resulting impacts on food safety (RONAFA) – adopted 01.12.2016', *EFSA* Journal 15/1 (2017), 4666.
2016	EFSA/ECDC. 'The European Union summary report on antimicrobial resistance in zoonotic and indicator bacteria from humans, animals and food in 2014', *EFSA Journal* 14/2 (2016), 4380.
2016	ECDC. *AMR surveillance in Europe in 2015* (Stockholm: ECDC, 2016).
2016	EC. 'Council conclusions on the next steps under a One Health approach to combat antimicrobial resistance', *Official Journal of the European Union*, C269/05, 23.07.2016.
2016	Smith, Elta et al., *Evaluation of the EC Action Plan against the rising threats from antimicrobial resistance* (Brussels: European Commission, 2016).
2016	EC. *Action Plan Against the rising threats from Antimicrobial Resistance: Road Map* (Brussels: European Commission, 2016).
2016	EMA. *CVMP strategy on antimicrobials 2016-2020* (London: EMA, 2016).
2016	EMA. *Sales of veterinary antimicrobial agents in 29 EU/EEA countries in 2014 (ESVAC report). Trends from 2011 to 2014* (London: EMA, 2016).
2016	WHO. *National antimicrobial resistance surveillance systems and participation in the Global Antimicrobial Resistance Surveillance System (GLASS) A guide to planning, implementation, and monitoring and evaluation* (Geneva: WHO, 2016).
2016	WHO FAO OIE. *A manual for developing national action plans* (2016).

2016	FAO. *Drivers and Dynamics and Epidemiology of Antimicrobial Resistance in Animal Production* (Rome: FAO, 2016).
2016	FAO. *Antimicrobial resistance and our food systems: challenges and solutions* (Rome: FAO, 2016).
2016	FAO. *Antimicrobial resistance. What is it* (Rome: FAO, 2016).
2016	FAO. *Antimicrobial resistance. Global commitment to local action* (Rome: FAO, 2016).
2016	FAO. *Update on FAOs work on AMR* (Rome: FAO, 2016).
2016	FAO. *The FAO action plan on antimicrobial resistance 2016-2020* (Rome: FAO, 2016).
2016	OIE. *OIE Annual report on the use of antimicrobial agents in animals - Better understanding of the global situation* (Paris: OIE, 2016).
2016	OIE. *Combatting Antimicrobial Resistance Through A One Health Approach: Actions and OIE Strategy* (Paris: OIE, 2016).
2016	OIE. *The OIE Strategy on Antimicrobial Resistance and the Prudent Use of Antimicrobials* (Paris: OIE, 2016).
2016	*Tackling Drug-Resistant Infections Globally: Final Report and Recommendations* (London: AMR Review, 2016)
2016	OECD. *Antimicrobial Resistance: Policy Insights* (Paris: OECD, 2016).
2016	TATFAR. *Economic incentives for antimicrobial therapy development: summary from the TATFAR* (2016).
2017	ICF. 'EU external scientific report Perceptions on the human health impact of antimicrobial resistance (AMR) and antibiotics use in animals across the EU', *EFSA supporting publication* (2017), EN-1183.
2017	TATFAR. 'Pull Incentives for Antibacterial Drug Development: An Analysis by the Transatlantic Task Force on Antimicrobial Resistance', *Clinical Infectious Diseases* 65/8 (2017), 1378-1382.
2017	G20. *Germany Hamburg G20 Leaders´ Declaration: Shaping an interconnected world* (Hamburg, 2017).
2017	G20. *Berlin Declaration of the G20 Health Ministers: Together Today for a Healthy Tomorrow* (Berlin, 2017).
2017	EMA/EFSA. 'EMA and EFSA Joint Scientific Opinion on measures to reduce the need to use antimicrobial agents in animal husbandry in the European Union, and the resulting impacts on food safety (RONAFA)', *EFSA Journal* 15/1 (2017), 4666.
2017	EC. *A European One Health Action Plan against Antimicrobial Resistance (AMR)* (Brussels: EC, 2017).
2017	EC. *Antimicrobial resistance and causes of non-prudent use of antibiotics in human medicine in the EU* (Brussels: EU, 2017).
2017	ECDC. *AMR surveillance in Europe in 2016* (Stockholm: ECDC, 2017).
2017	ECDC. *EU Guidelines for the prudent use of antimicrobials in human health* (Stockholm: ECDC, 2017).
2017	ECDC, EFSA and EMA. 'Joint Scientific Opinion on a list of outcome indicators as regards surveillance of antimicrobial resistance and antimicrobial consumption in humans and food-producing animals', *EFSA Journal* 15/10 (2017), 5017.

2017	ECDC/EFSA/EMA. 'Second joint report on the integrated analysis of the consumption of antimicrobial agents and occurrence of antimicrobial resistance in bacteria from humans and food-producing animals (JIACRA 2)', *EFSA Journal* 15/7 (2017), 4872.
2017	ECDC/EFSA. 'The European Union summary report on antimicrobial resistance in zoonotic and indicator bacteria from humans, animals and food in 2015', *EFSA Journal* 15/2 (2017), 4694.
2017	World Bank. *Assessing the Global economic and poverty effects of antimicrobial resistance* [Policy Research Working Paper; No. 8133] (Washington DC: World Bank, 2017).
2017	World Bank. *Drug Resistant Infections: A Threat to our Economic Future* (Washington DC: World Bank, 2017).
2017	EMA. *Sales of veterinary antimicrobial agents in 30 EU/EEA countries in 2015 (ESVAC report). Trends from 2010 to 2015* (London: EMA, 2017).
2017	WHO. *Guidelines for the prevention and control of carbapenem-resistant Enterobacteriaceae, Acinetobacter baumannii and Pseudomonas aeruginosa in health care facilities* (Geneva: World Health Organization, 2017).
2017	WHO. *Prioritization of pathogens to guide discovery, research and development of new antibiotics for drug-resistant bacterial infections, including tuberculosis* (Geneva: World Health Organization, 2017).
2017	WHO. *Integrated Surveillance of Antimicrobial Resistance in Foodborne Bacteria: Application of a One Health Approach* (Geneva: World Health Organization, 2017).
2017	WHO. *Critically important antimicrobials for human medicine – 5th rev*. (Geneva: World Health Organization, 2017).
2017	WHO. *Global Priority List of Antibiotic-Resistant Bacteria To Guide Research, Discovery and Development of new Antibiotics* (Geneva: WHO, 2017).
2017	WHO. *Antibacterial agents in clinical development – an analysis of the antibacterial clinical development pipeline, including Mycobacterium tuberculosis* (Geneva: WHO, 2017).
2017	ECA/FAO. *Antimicrobial resistance (AMR): the loss of a major defence to the emerging challenge?* 40th Session. Budapest, Hungary. 27-28 September 2017 (Rome: ECA/FAO, 2017)
2017	FAO. *Antimicrobial resistance in food and agriculture* (Rome: FAO, 2017).
2017	FAO. *Antimicrobial resistance a serious threat to human and animal health.* Available at http://www.fao.org/3/i6370e/i6370e.pdf (Rome: FAO, 2017).
2017	FAO. *FAO initiatives on prevention and control of AMR.* Available at http://www.fao.org/3/i7118e/i7118e.pdf (Rome: FAO, 2017).
2017	FAO, WHO, OIE. *Country Progress in the implementation of the global action plan on antimicrobial resistance: WHO, FAO and OIE Launch Global Tripartite Database* (2017).
2017	FAO, WHO, OIE. *Monitoring and evaluation of the Global Action Plan on Antimicrobial Resistance*: *Proposed approach*, August 2017
2017	OECD, WHO, FAO, OIE. *Tackling Antimicrobial Resistance. Ensuring Sustainable R&D* (2017)

2017	OECD. 'Low-value health care with high stakes: Promoting the rational use of antimicrobials', in *Tackling Wasteful Spending on Health* (Paris: OECD, 2019).
2018	FAO, WHO, OIE. *Memorandum of understanding between FAO, OIE, and WHO*. (2018).
2018	EC. *Measures to Tackle Antimicrobial Resistance Through the Prudent Use of Antimicrobials in Animals* (Luxembourg: EU, 2018).
2018	EC. *Non-EU Countries' National Policies and Measures on Antimicrobial Resistance* (Luxembourg: EU, 2018).
2018	EFSA/ECDC. 'The European Union Summary Report on antimicrobial resistance in zoonotic and indicator bacteria from humans, animals and food in 2016', *EFSA Journal* 16/2 (2018), 5182.
2018	OECD. *Stemming the Superbug Tide, Just A Few Dollars More* (Paris: OECD, 2018).
2018	European Parliament. *Report on a European One Health Action Plan against Antimicrobial Resistance.* Available at https://www.europarl.europa.eu/doceo/document/A-8-2018-0257_EN.pdf (2018).
2018	ECDC. *Annual Epidemiological Report for 2014 Antimicrobial consumption* (Stockholm: ECDC, 2018).
2018	ECDC. *Annual Epidemiological Report for 2015 Antimicrobial consumption* (Stockholm: ECDC, 2018).
2018	ECDC. *Annual Epidemiological Report for 2016 Antimicrobial consumption* (Stockholm: ECDC, 2018).
2018	ECDC. *AMR surveillance in Europe in 2017* (Stockholm: ECDC, 2018).
2018	ECDC. *Annual Epidemiological Report for 2017 Antimicrobial consumption* (Stockholm: ECDC, 2018)
2018	ECDC. *Surveillance of antimicrobial consumption in Europe in 2013-2014* (Stockholm: ECDC, 2018)
2018	EMA. *Sales of veterinary antimicrobial agents in 30 EU/EEA countries in 2016 (ESVAC report). Trends from 2010 to 2016* (London: EMA, 2018).
2018	WHO, FAO, OIE. *Monitoring Global Progress On Addressing Antimicrobial Resistance. Analysis report of the second round of results of AMR country-self-assessment survey* (Geneva: WHO, 2018).
2018	WHO. *WHO report on surveillance of antibiotic consumption: 2016-2018 early implementation* (Geneva: World Health Organization, 2018).
2018	WHO. *Global antimicrobial resistance surveillance system (GLASS) report: early implementation 2016-2017* (Geneva: WHO, 2018).
2018	WHO. *Critically Important Antimicrobials for Human Medicine: 6th revision* (Geneva: WHO, 2018).
2018	WHO. *Update of antibacterial agents in clinical development* (Geneva: WHO, 2018).
2018	WHO. *Tackling Antimicrobial Resistance (AMR) together. Working Paper 5.0: Enhancing the focus on gender and equity* (Geneva: WHO, 2018).
2018	WHO. *Tackling Antimicrobial Resistance (AMR) together. Working Paper 1.0: Multisectorial coordination* (Geneva: WHO, 2018).

2018	FAO. *FAO initiatives on prevention and control of antimicrobial resistance: Addressing antimicrobial usage in Asia's livestock production industry* (Rome: FAO, 2018).
2018	FAO. *FAO Information Pack Supporting national efforts to minimise risks from Antimicrobial Resistance (AMR)* (Rome: FAO, 2018).
2018	FAO/RAP. *Antimicrobial resistance policy review and development framework* (Bangkok: FAO/RAP, 2018).
2018	Welcome, US CDC, *UK Science and Innovation Network. Initiatives for Addressing Antimicrobial Resistance in the Environment* (2018).
2018	OIE. *Annual Report on antimicrobial agents intending for use in animals – Better Understanding the situation (Third Edition)* (Paris: OIE, 2018).
2019	D'Atri et al (TATFAR). 'Targets for the reduction of antibiotic use in humans in the Transatlantic Taskforce on Antimicrobial Resistance (TATFAR) partner countries', *Eurosurveillance* 24/28 (2019).
2019	TATFAR. *Data for Action: Using Available Data Sources to Track Antibiotic Use in your Country* (2019).
2019	UNICEF. *Time is running out Technical note on antimicrobial resistance* (New York: UNICEF, 2019)
2019	IACG. *No Time to wait: securing the future from drug-resistant infections* (UN, 2019).
2019	WHO/ European Observatory on Health Systems and Policies. *Averting the AMR crisis. Policy Brief 32. What are the avenues for policy action for countries in Europe?* (Copenhagen: WHO Regional Office for Europe, 2019).
2019	WHO. *Antibacterial agents in preclinical development: an open access database* (Geneva: WHO, 2019).
2019	FAO and WHO. *Joint FAO/WHO Expert Meeting in collaboration with OIE on Foodborne Antimicrobial Resistance: Role of the Environment, Crops and Biocides – Meeting report. Microbiological Risk Assessment Series no. 34.* (Rome: FAO/WHO, 2019).
2019	WHO. *Antibacterial agents in clinical development: an analysis of the antibacterial clinical development pipeline* (Geneva: WHO, 2019).
2019	WHO. *Antimicrobial stewardship programmes in health-care facilities in low- and middle-income countries. A practical toolkit* (Geneva: WHO, 2019).
2019	WHO. *Meeting Report Antibiotic Shortages: Magnitude, Causes and Possible Solutions. Norwegian Directorate of Health, Oslo, Norway 10-11 December 2018* (Geneva: WHO, 2019).
2019	WHO. *Meeting report for technical consultation on in vitro diagnostics for AMR, 27–28 March 2019* (Geneva: WHO, 2019).
2019	WHO. *Landscape of diagnostics against antibacterial resistance, gaps and priorities* (Geneva: WHO, 2019).
2019	WHO, FAO, OIE. *Monitoring and Evaluation of the Global Action Plan on Antimicrobial Resistance Framework and recommended indicators* (2019).
2019	WHO. *Turning plans into action for antimicrobial resistance (AMR). Working paper 2.0: implementation and coordination* (Geneva: WHO, 2019).
2019	FAO. *Stakeholder Consultation on an FAO Database for Antimicrobial Resistance (AMR) in Food and Agriculture* (Rome: FAO, 2019).

2019	FAO. *Monitoring and surveillance of antimicrobial resistance in bacteria from healthy food animals intended for consumption* [Vol. 1] (Bangkok: FAO, 2019).
2019	OIE. *Report of the meeting of the OIE working group on Antimicrobial Resistance. Paris, October 1-3, 2019* (Paris: OIE, 2019).
2019	World Bank. *Pulling Together to Beat Superbugs: Knowledge and Implementation Gaps in Addressing Antimicrobial Resistance* (Washington DC: World Bank, 2019).
2019	FAO. *Antimicrobial Resistance (Draft Resolution)* - C 2019/LIM/22 (2019).
2019	FAO and Ministry of Food and Environment of Denmark. *Tackling Antimicrobial Use and Resistance in Pig Production. Lessons Learned in Denmark* (Rome: FAO, 2019).
2019	FAO RAP. *Managing antimicrobial resistance in Thailand* (2019).
2019	FAO, WHO, OIE. *Antimicrobial Resistance Multi-Partner Trust Fund* (2019).
2019	EMA. *Sales of veterinary antimicrobial agents in 31 European countries in 2017 Trends from 2010 to 2017* (Amsterdam: EMA, 2019).
2019	ECDC. *Antimicrobial consumption in the EU/EEA Annual epidemiological report for 2018* (Stockholm: ECDC, 2019).
2019	ECDC. *AMR surveillance in Europe in 2018* (Stockholm: ECDC, 2019).
2019	ECDC and OECD. *Antimicrobial Resistance Tackling the Burden in the European Union* (2019).
2020	WHO. *GLASS whole-genome sequencing for surveillance of antimicrobial resistance* (Geneva: WHO, 2020).
2020	WHO. *GLASS guidance for national reference laboratories* (Geneva: WHO, 2020).
2020	WHO. *First meeting of the WHO Antifungal Expert Group on Identifying Priority Fungal Pathogens: meeting report* (Geneva: WHO, 2020).
2020	WHO. *A financial model for an impact investment fund for the development of antibacterial treatments and diagnostics: a user guide* (Geneva: WHO, 2020.)
2020	WHO. *GLASS method for estimating attributable mortality of antimicrobial resistant bloodstream infections* (Geneva: WHO, 2020).
2020	WHO. *Global antimicrobial resistance surveillance system (GLASS) report: early implementation 2020* (Geneva: WHO, 2020).
2020	WHO. *Target product profiles for needed antibacterial agents: enteric fever, gonorrhoea, neonatal sepsis, urinary tract infections and meeting report* (Geneva: WHO, 2020).
2020	WHO. *Target product profiles for antibacterial resistance diagnostics. Geneva: World Health Organization* (Geneva: WHO, 2020).
2020	WHO, FAO, OIE. *Technical brief on water, sanitation, hygiene and wastewater management to prevent infections and reduce the spread of antimicrobial resistance* (WHO/FAO/OIE 2020).
2020	FAO, OIE, WHO. *International instruments on the use of antimicrobials across the human, animal and plant sectors* (2020).
2020	Bondad-Reataso et al. [FAO], ‘Outputs and Activities of FAO Project FMM/RAS/298/MULON Antimicrobial Resistance in Fisheries and Summary of FAO’s Recent Work on Antimicrobial Resistance in Aquaculture’, *FAO Fisheries and Aquaculture Circular* No. 1215. Rome, FAO.

2020	FAO. *FAO Expert Working Group Meeting 'Scoping Exercise To Increase The Understanding Of Risks of Antimicrobial Resistance (AMR) in Aquaculture'* [held in Palermo, Italy, 2018] (2020)
2020	FAO *Antimicrobial resistance in food.* Available at http://www.fao.org/3/ca8275en/CA8275EN.pdf (March 2020).
2020	FAO. *Progress report on the implementation of FAO Action Plan on Antimicrobial Resistance (AMR) 2016-2020, and the proposal for a new FAO Action Plan on AMR 2021-2025* [COAG/2020/8] (Rome: FAO, 2020).
2020	FAO/APRC. *Regional conference for Asia and the Pacific. Report on Antimicrobial Resistance (AMR): Bhutan 17-20 February* (Bangkok: FAO/APRC, 2020).
2020	FAO. *Antimicrobial Resistance (AMR) in relation to pesticide use in plant production* (2020).
2020	FAO. 'Improving communications for antimicrobial resistance (AMR) in Africa: How should we move forward?' *FSN Forum in Africa* – summary of online discussion no. 16 (2020).
2020	FAO. *Tackling Antimicrobial Use And Resistance in Dairy Cattle. Lessons Learned in Sweden* (Rome: FAO, 2020)
2020	OIE. *Annual report on antimicrobials intended for use in animals. Better understanding of the global situation* (Paris: OIE, 2020).
2020	OIE. *Report of the Meeting of the OIE Working Group on Antimicrobial Resistance April 2020* (Paris: OIE, 2020).
2020	OIE. *Report of the meeting of the OIE working group on Antimicrobial Resistance. Paris (via zoom), October 13-15, 2020* (Paris: OIE, 2020).
2020	EU. *Progress Report 2017 EU AMR Action Plan* (update for Q2 2019) (2020).
2020	ECDC. *Antimicrobial consumption in the EU/EEA Annual epidemiological report for 2019* (Stockholm: ECDC, 2020).
2020	ECDC. *Antimicrobial resistance in the EU/EEA (EARS-Net) Annual Epidemiological Report for 2019* (Stockholm: ECDC, 2020).
2020	EMA. *Sales of veterinary antimicrobial agents in 31 European countries in 2018 Trends from 2010 to 2018* (Amsterdam: EMA, 2020).
