# Supplementary material for: Waves of attention: patterns and themes of international antimicrobial resistance reports, 1945–2020
Source: BMJ Glob Health. 2021 Nov 5;6(11):e006909. doi: 10.1136/bmjgh-2021-006909 (PMC8573652; doi:10.1136/bmjgh-2021-006909)
Supplement: Supplementary data [file bmjgh-2021-006909supp003.pdf]

**Supplementary Table Three: Consensus Reports**

| Year | Title of Report |
|---|---|
| 1957 | American Medical Association (AMA). (November 14, 1957). *Conference on Staphylococcic Infections: Staphylococcic Infections in the Hospital and Community.* Cleveland, Ohio. |
| 1958 | United States Public Health Service (USPHS), National Academy of Sciences-National Research Council (NAS-NRC). (1958). *The National Conference on Hospital-Acquired Staphylococcal Disease.* Atlanta, Georgia. September 15-17, |
| 1961 | World Health Organisation (WHO). (1961). Standardization of methods for conducting microbic sensitivity tests: second report of the Expert Committee on Antibiotics [meeting held in Geneva from 11 to 16 July 1960]. |
| 1962 | United States Food and Drug Administration (US FDA) (1962). 'The Kefauver-Harris Drug Amendments' Drug Amendments Act of October 10, 1962, Public Law 87-781, 76 STAT 780. Washington. |
| 1969 | Swann, M., Baxter K., Field H., et al. (1969). Report of the Joint Committee on the use of Antibiotics in Animal Husbandry and Veterinary Medicine. HMSO: London. |
| 1971 | Ericsson, H. & Sherris, J. (1971). Antibiotic sensitivity testing. Report of an international collaborative study. Acta pathologica et microbiologica scandinavica. Suppl. 217:90. |
| 1973 | US Food and Drug Administration (US FDA). (1973). FDA Task Force on the Clinical Use of Antibiotics. FDA: Washington. |
| 1973 | World Health Organisation. (WHO). (1973). Control of Harmful Residues in Food for Human and Animal Consumption: The Public Health Aspects of Antibiotics in Feedstuffs. Report of a Working Group. Bremen. 1-5 October. |
| 1977 | World Health Organisation (WHO). (1977). The Selection of Essential Drugs. Geneva. |
| 1978 | World Health Organisation (WHO). (1978). Surveillance for the prevention and control of health hazards due to antibiotic-resistant enterobacteria: report of a WHO meeting [held in Geneva from 18 to 24 October 1977]. |
| 1981 | Levy, S., Clowes, R. and Koenig, E. (conveners) (1981). Statement Regarding Worldwide Antibiotic Misuse. Molecular Biology, Pathogenicity, and Ecology of Bacterial Plasmids Conference. Plenum: Santo Domingo |
| 1985 | Lag (1985). Om Foder. Act SFS 1985:295 (Legislation). Swedish Act relative to feeding stuffs, banning the use of AGP in agriculture. |
| 1987 | Kunin, C., Lipton, H., Tupasi, T. et al. (1987). Social, behavioural, and practical factors affecting antibiotic use worldwide: report of NIH Task Force 4. *Reviews of infectious diseases*, 9(3):S270-S285. |
| 1992 | Shope, R., and Oaks, S. (1992). Emerging Infections: Microbial Threats to Health in the United States. Pp294. National Academies Press: Washington. |
| 1994 | The Genesis Report. (1994). The real war on drugs: Bacteria are winning. New Jersey. |
| 1995 | Mochales, F., Castelló, R., Marqués, J. et al. (1995). Resistencia Microbiana: Iqué Hacer? Informe del panel de expertos.[Microbial Resistance: What to do? Expert Panel Report]. Rev Esp Salus Pública. 69(6):445-461. |
| 1998 | Rosdahl, V., and Pedersen, K. (1998). The Copenhagen recommendations. In Report from the European Union Conference on "the Microbial Threat". Ministry of Health and Ministry of Food, Agriculture and Fisheries: Denmark. |
| 2001 | World Health Organisation (WHO). (2001). Global Strategy for Containment of Antimicrobial Resistance. No. WHO/CDS/CSR/DRS/2001.2). WHO: Geneva. |
| 2003 | European Parliament (2003). Regulation (EC) No 1831/2003 of the European Parliament and the Council of 22 September 2003 on additives for use in animal nutrition. *Official Journal of the European Union*, L268/29, 18.10.2003. |

| | |
|---|---|
| 2004 | The Infectious Diseases Society of America (IDSA). (2004). Bad Bugs, No Drugs. As Antibiotic Discovery Stagnates…A Public Health Crisis Brews. IDSA: Virginia. |
| 2005 | Europe Union Commission (EUC) (22 December, 2005). Ban on antibiotics as growth promoters in animal feed enters into effect. Regulation 1831/2003/EC on additives for use in animal nutrition, replacing Directive 70/524/EEC on additives in feed-stuffs. EUC: Brussels. |
| 2007 | European Medicines Agency (EMA). (2007). Committee for Medical Products for Veterinarian use - rules of procedure. EMEA/CVMP/422/04- Rev. 1, EMEA/ MB/ 47098/ 2007. EMA: London. |
| 2009 | European Centres for Disease Prevention and Control (ECDP) and European Medicines Agency (EMA). (2009). The bacterial challenge–time to react a call to narrow the gap between multidrug-resistant bacteria in the EU and development of new antibacterial agents. Solna: ECDC & EMEA Joint Press Release. |
| 2011 | World Health Organisation (WHO). (2011). Jaipur declaration on antimicrobial resistance. WHO Regional Office for South-East Asia: Jaipur, India. |
| 2011 | European Commission. (2011). Communication from the Commission to the European Parliament and the Council. Action plan against the rising threats from antimicrobial resistance. COM(2011) 748 final. European Commission: Brussels. |
| 2011 | Bundesministerium für Gesundheit, gemeinsam mit Bundesministerium für Ernährung, Landwirtschaft und Verbraucherschutz; und Bundesministerium für Bildung und Forschung (2011): DART - Deutsche Antibiotika-Resistenzstrategie, Berlin [Federal Ministry of Health, together with the Federal Ministry for Food, Agriculture and Consumer Protection; and the Federal Ministry of Education and Research. (2011): DART - German Antibiotic Resistance Strategy, Berlin]. |
| 2012 | Council of the European Union. (2012). Council conclusion on the impact of antimicrobial resistance in the human health sector and in the veterinary sector - a 'One Health' perspective. 3177th Employment, Social Policy, Health And Consumer Affairs Council Meeting Luxembourg, 22 June 2012. Council of the European Union: Brussels. |
| 2012 | World Health Organisation (WHO). (2012). The evolving threat of antimicrobial resistance: options for action. WHO: Geneva. |
| 2012 | European Parliament Committee on the Environment, Public Health and Food Safety. (2012). Report on the Microbial Challenge - Rising threats from Antimicrobial Resistance (2012/2041 (INI). Doc. A7-0373/2012. European Parliament: Brussels. |
| 2013 | Centers for Disease Control and Prevention (CDCP). (2013). Antibiotic resistance threats in the United States. CDC: Atlanta. |
| 2013 | European Commission. (2013): Antimicrobial Resistance, Special Eurobarometer 407 Report. European Commission: Brussels. |
| 2013 | Davies, S. (2013). Annual Report of the Chief Medical Officer, Volume 2 (The Drugs Don't Work). Department of Health: London. |
| 2014 | Antibiotic Resistance Coalition (ARC) (2014). Civil Society Declaration on Antibiotic Resistance. ARC (John Hopkins School of Public Health): Baltimore. |
| 2014 | European Medicine Agency (EMA). (2014). Sales of veterinary antimicrobial agents in 26 EU/EEA countries in 2012. Fourth ESVAC report. (EMA/333921/2014). EMA: Amsterdam. |
| 2014 | Fødevarestyrelsen. (2014): MRSA risikovurdering Udfærdiget af MRSA-ekspertgruppen. Fødevarestyrelsen: København. [The Danish Veterinary and Food Administration (2014). MRSA risk assessment. DVFA: Copenhagen]. |
| 2014 | World Health Organisation (WHO). (2014). Antimicrobial Resistance: Global Report on Surveillance. WHO: Geneva. |

| | |
|---|---|
| 2015 | Public Health England (PHE). (2015). Start Smart - Then Focus. Antimicrobial Stewardship Toolkit for English Hospitals. [An update of the 2011 guidance]. PHE: London. |
| 2015 | European Centre for Disease Prevention and Control (ECDPC). (2015). Antimicrobial resistance surveillance in Europe 2014. Annual Report of the European Antimicrobial Resistance Surveillance Network (EARS-Net). ECDPC: Stockholm. |
| 2015 | World Health Organisation (WHO). (2015). Global Action Plan on Antimicrobial Resistance. WHO: Geneva |
| 2016 | United Nations (UN) General Assembly. (2016). Political Declaration of the High-level Meeting of the General Assembly on Antimicrobial Resistance, Proceedings of the 71st Session, Agenda Item 127 A/71/L.2. 22 September, 2016. UN: New York. |
| 2016 | O'Neill, J. (2016). Tackling Drug-Resistant Infections Globally: Final Report and Recommendations. Government of the United Kingdom: UK. |
| 2016 | World Organisation for Animal Health (OIE). (2016). The OIE Strategy on Antimicrobial Resistance and the Prudent Use of Antimicrobials. OIE: Paris. |
| 2016 | AMR Industry Alliance (2016). Declaration on Combating Antimicrobial Resistance. AMR Industry Alliance: Geneva. |
| 2016 | Changing Markets Foundation and Ecostorm. (2016). Superbugs in the Supply Chain: How pollution from antibiotics factories in India and China is fuelling the global rise of drug-resistant infections. Changing Markets Foundation: Utrecht. |
| 2017 | Jonas, O., Irwin, A., Berthe, F., Le Gall, F, & Marquez, P. (2017). Final report. Drug-resistant infections: a threat to our economic future: Vol. 2. HNP/Agriculture Global Antimicrobial Resistance Initiative Washington. World Bank Group: Washington. |
| 2017 | European Centre for Disease Prevention and Control (ECDC), European Food Safety Authority (EFSA) and European Medicines Agency (EMA). (2017). ECDC/EFSA/EMA second joint report on the integrated analysis of the consumption of antimicrobial agents and occurrence of antimicrobial resistance in bacteria from humans and food-producing animals. *EFSA Journal*, 15(7): 4872. |
| 2017 | World Bank (2017). Drug-resistant Infections: A Threat to Our Economic Future. Washington DC: World Bank Group. |
| 2018 | Organisation for Economic Co-operation and Development (OECD). (2018). Stemming the Superbug Tide: Just a Few Dollars More, OECD Health Policy Studies. OECD Publishing: Paris. |
| 2018 | Tacconelli, E., Carrara, E., Savoldi, A., et al. (2018). Discovery, research, and development of new antibiotics: the WHO priority list of antibiotic-resistant bacteria and tuberculosis. *The Lancet Infectious Diseases*, 18(3), 318-327. |
| 2018 | World Health Organisation (WHO). (2018). Global antimicrobial resistance surveillance system (GLASS) report. Early implementation 2016-2017. WHO: Geneva. |
| 2018 | Wellcome Trust. (2018). Initiatives for addressing Antimicrobial Resistance in the Environment. Wellcome Trust: London. |
| 2019 | UN Interagency Coordination Group (IACG) on Antimicrobial Resistance. (2019). No Time to Wait: Securing the future from drug-resistant infections. Report to the Secretary-General of the United Nations. IACG: New York. |
| 2019 | Council of Canadian Academies (CCA). (2019). When Antibiotics Fail. The Expert Panel on the Potential Socio-Economic Impacts of Antimicrobial Resistance in Canada. CCA: Ottawa. |

| | |
|---|---|
| 2019 | U.S. Department of Health and Human Services, Centers for Disease Control and Prevention (CDCP). (2019). Antibiotic Resistance Threats in The United States. Department of Health and Human Services. CDCP: Atlanta. |
| 2019 | World Health Organisation (WHO). (2019). Global antimicrobial resistance surveillance system (GLASS) report. Early implementation 2017-2018. WHO: Geneva. |
| 2019 | World Health Organisation (WHO). (2019). Antibacterial Agents in Clinical Development. WHO: Geneva. |
| 2019 | The Boston Consulting Group (BCG), Wellcome Trust (2019). Vaccines to tackle drug resistant infections. An evaluation of R&D opportunities. BCG, Wellcome Trust: London. |
| 2019 | Chatham House, The Royal Institute of International Affairs (2019). Review on progress (full report). Chatham House: London. |
| 2019 | The Pew Charitable Trusts. (2019). A Scientific Roadmap for Antibiotic Discovery: A sustained and robust pipeline of new antibacterial drugs and therapies is critical to preserve public health. The Pew Charitable Trusts: London. |
| 2019 | Hall, W., McDonnell, O'Neill (2019). Superbugs: An arms race against bacteria. Harvard University Press. |
| 2020 | World Health Organisation (WHO). (2020). Global antimicrobial resistance and use surveillance system (GLASS) report. Early implementation 2020. WHO: Geneva. |
